# Galectin-3 and prohibitin 1 are autoantigens in IgG4-related cholangitis without clear-cut protective effects against toxic bile acids

**DOI:** 10.3389/fimmu.2023.1251134

**Published:** 2024-01-25

**Authors:** Remco Kersten, David C. Trampert, Lowiek M. Hubers, Dagmar Tolenaars, Harmjan R. Vos, Stan F. J. van de Graaf, Ulrich Beuers

**Affiliations:** ^1^ Tytgat Institute for Liver and Intestinal Research, Department of Gastroenterology and Hepatology, Amsterdam Gastroenterology Endocrinology Metabolism (AGEM), Amsterdam University Medical Center (UMC), University of Amsterdam, Amsterdam, Netherlands; ^2^ Oncode Institute and Molecular Cancer Research, Center for Molecular Medicine, University Medical Center Utrecht, Utrecht, Netherlands

**Keywords:** biliary bicarbonate umbrella, cholangiopathy, cholestasis, IgG4-related systemic disease, immune-mediated disease, secretory organs

## Abstract

**Background and aims:**

IgG4-related cholangitis (IRC) is the hepatobiliary manifestation of IgG4-related disease, a systemic B cell-driven fibro-inflammatory disorder. Four autoantigens have recently been described in IgG4-RD: annexin A11, galectin-3, laminin 511-E8, and prohibitin 1. We have previously reported a protective role of annexin A11 and laminin 511-E8 in human cholangiocytes against toxic bile acids. Here, we explored the potentially protective role of the carbohydrate-binding lectin galectin-3 and the scaffold proteins prohibitins 1 and 2.

**Methods:**

Anti-galectin-3, anti-prohibitin 1 and 2 autoantibody positivity in IRC and healthy and disease (primary sclerosing cholangitis (PSC)) control sera was assessed by ELISA/liquid chromatography–tandem mass spectrometry (LC-MS/MS). Human H69 cholangiocytes were subjected to short hairpin RNA (shRNA) knockdown targeting galectin-3 (*LGALS3*), prohibitin 1 (*PHB1*), and prohibitin 2 (*PHB2*). H69 cholangiocytes were also exposed to recombinant galectin-3, the inhibitor GB1107, recombinant prohibitin 1, and the pan-prohibitin inhibitor rocaglamide. Protection against bile acid toxicity was assessed by intracellular pH (pH_i_) measurements using BCECF-AM, 22,23-^3^H-glycochenodeoxycholic acid (^3^H-GCDC) influx, and GCDC-induced apoptosis using Caspase-3/7 assays.

**Results:**

Anti-galectin-3 autoantibodies were detected in 13.5% of individuals with IRC but not in PSC. Knockdown of *LGALS3* and galectin-3 inhibition with GB1107 did not affect pH_i_, whereas recombinant galectin-3 incubation lowered pH_i_. *LGALS3* knockdown increased GCDC-influx but not GCDC-induced apoptosis. GB1107 reduced GCDC-influx and GCDC-induced apoptosis. Recombinant galectin-3 tended to decrease GCDC-influx and GCDC-induced apoptosis. Anti-prohibitin 1 autoantibodies were detected in 61.5% and 35.7% of individuals with IRC and PSC, respectively. Knockdown of *PHB1*, combined *PHB1/2* KD, treatment with rocaglamide, and recombinant prohibitin 1 all lowered pH_i_. Knockdown of *PHB1*, *PHB2*, or combined *PHB1/2* did not alter GCDC-influx, yet knockdown of *PHB1* increased GCDC-induced apoptosis. Conversely, rocaglamide reduced GCDC-influx but did not attenuate GCDC-induced apoptosis. Recombinant prohibitin 1 did not affect GCDC-influx or GCDC-induced apoptosis. Finally, anti-galectin-3 and anti-prohibitin 1 autoantibody pretreatment did not lead to increased GCDC-influx.

**Conclusions:**

A subset of individuals with IRC have autoantibodies against galectin-3 and prohibitin 1. Gene-specific knockdown, pharmacological inhibition, and recombinant protein substitution did not clearly disclose a protective role of these autoantigens in human cholangiocytes against toxic bile acids. The involvement of these autoantibodies in processes surpassing epithelial secretion remains to be elucidated.

## 1 Introduction

IgG4-related disease (IgG4-RD) is a systemic fibroinflammatory disorder that can affect many different organs, with the pancreatic and hepatobiliary manifestations being the most common, termed type 1 autoimmune pancreatitis (AIP) and IgG4-related cholangitis (IRC) (1). Serum IgG4 levels are often elevated in individuals with IRC (70%–90%) (1–3), and affected tissues are characterized by typical histopathological findings such as storiform fibrosis, obliterative phlebitis, and a predominantly lymphoplasmacytic infiltrate enriched with IgG4^+^ B cells.

The clinical presentation and management of IRC have several challenges. First, IRC is often misdiagnosed for either primary sclerosing cholangitis (PSC) or cholangiocarcinoma (CCA), leading to unjustified major hepatobiliary surgeries in up to 30% of individuals with IRC (4). Although an elevated level of serum IgG4 can aid in making the diagnosis of IRC, it is hampered by a lack of sensitivity and specificity (5). Second, subjects with IRC often require long-term corticosteroid therapy, as relapse occurs often upon steroid withdrawal (6). A better understanding of the pathophysiology of IRC would aid in both the diagnosis and treatment of individuals with IRC.

Currently, the pathophysiology of IRC remains unresolved. Our group and others have previously demonstrated the presence of dominant affinity matured IgG4^+^ B-cell receptor clones and clonally expanded plasmablasts in people with IRC and multiorgan IgG4-RD (7–9). These findings were highly suggestive of the presence of specific autoantigens in IgG4-RD and indeed were followed by the discovery of the autoantigens annexin A11, laminin 511-E8, galectin-3, and prohibitin 1 in people with IgG4-RD (10–13). The presence of autoantibodies against more than one of these four antigens has been demonstrated to correlate with disease severity, and autoantibody titers decreased upon successful treatment (11, 12, 14). Autoantibodies directed against these antigens were mainly of the IgG4 and IgG1 subclasses, but the potential pathogenicity of individual IgG1 or IgG4 autoantibodies in IgG4-RD has not been fully clarified. Mice exposed to isolated IgG1 or IgG4 from people with IgG-RD developed typical organ lesions (15). Notably, IgG1 was shown to be more destructive than IgG4, and the pathogenicity of patient IgG1 was reduced by simultaneous injection of patient IgG4 in a pre-clinical model system. In line with these findings, we have previously shown that two linear epitopes on the autoantigen annexin A11 were recognized by both IgG1 and IgG4 autoantibodies and that IgG4 competitively blocked the binding of IgG1 to these epitopes on annexin A11 (10). These findings indicate that IgG4 could potentially repress an IgG1-driven immune response in IgG4-RD, as also seen in other inflammatory conditions (16). At present, it is unclear whether autoantibodies detected in IgG4-RD are the main driver of IgG4-RD, make up additional pathogenic pathways, or just represent a non-pathogenic epiphenomenon. Nevertheless, there are autoimmune diseases where IgG4 autoantibodies are directly pathogenic (17–21). The potential pathogenicity of IgG1 or IgG4 autoantibodies in IRC could be driven by two mechanisms: i) via a direct functional effect of autoantibodies on the targeted autoantigens, or ii) via an indirect mechanism where autoantibody binding elicits an excessive immune response that would lead to obstructive cholestasis.

To address the potential direct pathogenicity of IgG1 and IgG4 autoantibodies in IRC, an in-depth understanding of the four targeted antigens is essential. To this end, our group has previously demonstrated that annexin A11 is involved in bicarbonate-rich fluid secretion (22). Annexin A11 was demonstrated to facilitate the trafficking of ANO1, a calcium-sensitive chloride channel, to the apical cholangiocyte membrane. This process was inhibited by incubating cholangiocytes with patient serum containing both IgG1 and IgG4 autoantibodies directed against annexin A11. ANO1 establishes a chloride gradient necessary for chloride/bicarbonate exchangers to secrete bicarbonate into the bile duct lumen at the juxta-apical surface of cholangiocytes. The formed apical alkaline layer, known as the biliary bicarbonate umbrella, keeps bile salts in their deprotonated, membrane-impermeable, and non-toxic state (23, 24). Dysfunction of the biliary bicarbonate umbrella leads to bile acids penetrating cholangiocytes and inducing cholangiocellular damage and death (24). Recent data on the role of the autoantigen laminin 511-E8 in cholangiocytes, among cell barrier functions, point toward the protection of cholangiocytes from toxic bile acids, potentially by stabilizing the biliary bicarbonate umbrella (25). The present work investigates the potential protective effect of the remaining autoantigens in IRC, galectin-3, and prohibitin 1.

**Figure 1:**
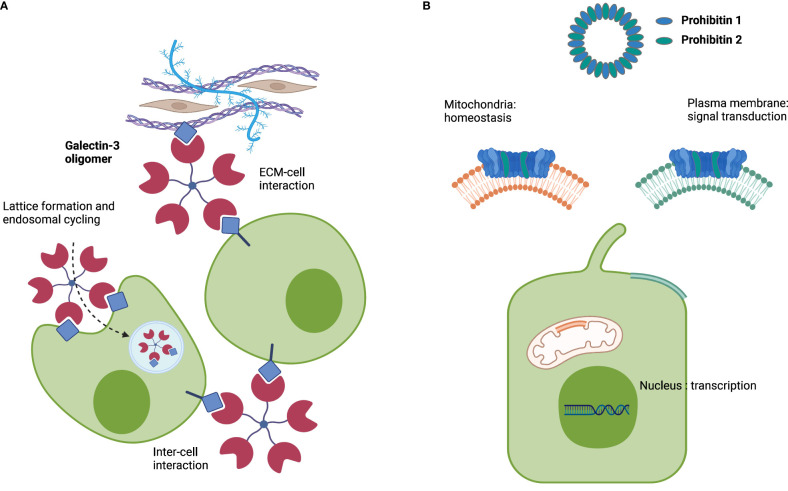
Schematic roles of galectin-3 and prohibitins 1 and 2. **(A)** Representation of the multifaceted roles of galectin-3. **(B)** Representation of the multifaceted roles of prohibitins 1 and 2 as a multimeric complex. Image created with BioRender.com.

Galectin-3 is a β-galactoside-binding lectin that forms pentamers. These pentamers facilitate extracellular matrix (ECM)–cell interactions and cell–cell interactions and form lattices at the plasma membrane of cells, followed by invagination and endosomal transport (**Figure 1A**) (26). Notably, galectin-3 is required for apical protein sorting, which takes place in a pH-dependent manner (27, 28). Autoantibodies directed against galectin-3 were first described in people with multiorgan IgG4-RD who predominantly presented with type 1 AIP and IgG4-related sialadenitis, two glandular organs that secrete bicarbonate-rich fluids (11). As the adequate formation of the biliary bicarbonate umbrella relies on the presence of various transporters and channels involved in bicarbonate secretion at the apical cholangiocyte membrane and a stable glycocalyx (23, 24), we speculated that galectin-3 could exert a protective effect against toxic bile acids in human cholangiocytes as previously described for the IRC autoantigens annexin A11 and laminin 511-E8 and that galectin-3 autoantibodies could antagonize this protective effect.

Prohibitins (PHBs) are scaffold proteins predominantly located in the inner mitochondrial membrane, where prohibitin 1 and prohibitin 2 together form a ring-like multimeric complex. Prohibitins also localize to the cytosol, nucleus, and plasma membrane, where they interact with cell membrane receptors, cytoskeletal transport systems, and transcription factors (**Figure 1B**) (29). Autoantibodies against prohibitin 1 were first described in people with dacryosialoadenopathy (Mikulicz’s disease), type 1 AIP, and retroperitoneal fibrosis, with predominant manifestations of IgG4-RD in bicarbonate-secreting glands (13). Notably, prohibitin 1 is downregulated in the liver tissue of people with primary biliary cholangitis (PBC) (30), an immune-mediated cholestatic liver disease where defective biliary bicarbonate secretion may play a pathogenic role (24, 31).

The present work first analyzed the presence of IgG4-RD autoantibodies against galectin-3 and prohibitins 1 and 2 in individuals with IRC in comparison to those with PSC (cholestatic disease control) and healthy volunteers. Second, the present work investigated the potential role of the autoantigens galectin-3 and prohibitins 1 and 2 in protecting human cholangiocytes against bile acid-induced toxicity.

## 2 Materials and methods

### 2.1 Human ethics statement

The use of patient serum samples and human liver tissue was approved by the local medical ethical committee in Amsterdam (MEC 10/007, 2022.0144, and 2020.081). Participants gave written informed consent prior to inclusion in the study.

### 2.2 Patients

Serum samples were obtained from people with IRC (n = 52), PSC (n = 15), CCA (n = 14), and healthy volunteers (n = 14). Diagnosis of IRC was made according to HISORt criteria (1, 6), whereas diagnosis of PSC was made according to the European Association for the Study of the Liver (EASL) clinical practice guidelines on sclerosing cholangitis (32). The diagnosis of CCA was made based on histological confirmation after suggestive imaging findings.

### 2.3 ELISA

Alternating rows of 96-well ELISA plates were coated with 100 µl of recombinant galectin-3 (5 µg/ml) (PeproTech #450-38), recombinant prohibitin 1 (1 µg/ml) (FineTest #P5113), recombinant annexin A11 (1 µg/ml) (Abcam #ab101050), or equivalent concentrations of bovine serum albumin (BSA) in phosphate-buffered saline (PBS) and incubated at 4°C overnight (11, 13). Wells were washed seven times with TBST pH 8.0 and subsequently blocked for 1 hour with 1% BSA/TBS pH 8.0 at room temperature. Wells were again washed seven times with TBST pH 8.0. Sera from people with IRC, PSC, CCA, or healthy controls were centrifuged at 9000 rcf for 20 minutes before wells were incubated with diluted serum (1:200 for galectin-3 or 1:20 for prohibitin 1 in 1% BSA/TBS pH 8.0) for 30 minutes at room temperature. After seven washes with TBST pH 8.0, wells were incubated with 100 µl of rabbit anti-human total IgG (Dako #P0214) antibody at 1:2000 dilution for 1 hour at room temperature. IgG4 (Southern Biotech #9200-05) and IgG1 (Southern Biotech #9052-05) specific autoantibodies were detected using secondary mouse anti-human antibodies directed at human IgG4 and human IgG1 at 1:2000 dilution. After seven washes, bound reactants were detected by incubation using 100 µl of 3,3′,5,5′-tetramethylbenzidine (TMB) chromogenic substrate (Merck #CL07). The reaction was subsequently stopped with 100 µl of stop solution (Thermo Scientific #N600), and absorbance was determined at 450 nm using the CLARIOstar (BMG LABTECH, Ortenberg, Germany). Samples were considered positive for autoantibodies against galectin-3, prohibitin 1, or annexin A11 when absorbance values were greater than the mean plus two standard deviations of the healthy control group as previously described (11, 14).

### 2.4 Immunoprecipitation and liquid chromatography–tandem mass spectrometry analysis

Immunoprecipitation and liquid chromatography–tandem mass spectrometry (LC-MS/MS) was performed as described previously (10). IgG4 high-affinity beads were washed two times with 0.1% NP-40/PBS and subsequently incubated with patient serum containing 600 µg of IgG4 for 1 hour at room temperature. The beads were then washed five times with 0.1% NP-40/PBS and transferred to new Eppendorf tubes. IgG4 antibodies were eluted twice for 10 minutes with citrate buffer pH 3.0. After centrifugation, the supernatant with eluted IgG4 was transferred and neutralized by 0.5 M borate buffer pH 8.8. Purified IgG4 was then covalently bound to tosyl-activated magnetic beads (Dynabeads M-280, Thermo Fisher Scientific) by incubating the beads overnight at 37°C in 125 µl of 3.6 M (NH_4_)_2_SO_4_/0.1 M borate buffer pH 8.8. Subsequently, beads were washed with 50 mM Tris pH 7.4, 1 M NaCl, and 1% NP-40 (w/v); blocked for 1 hour in 1 M Tris pH 7.4; washed again four times; and transferred to new Eppendorf tubes. Beads at a volume of 50 µl were then incubated overnight at 4°C with 1200 µl of H69 cytosolic cell lysate with the addition of 1 M NaCl and 1% (w/v) NP-40. Unbound proteins were removed by washing three times with 50 mM Tris pH 7.4, 1 M NaCl, and 1% NP-40 (w/v) and three times with PBS. During the washing process, the beads were transferred twice to a new Eppendorf tube. After washing, the supernatant was discarded, and the beads were frozen for LC-MS/MS analysis. Precipitated proteins were denatured using 8 M urea in 1 M NH_4_HCO_3_ reduced with 10 mM tris(2-carboxyethyl)phosphine at room temperature for 30 minutes. Hereafter, cysteines were alkylated with 40 mM chloroacetamide for 30 minutes. After diluting four times with 1 M ammonium bicarbonate, proteins were digested by 150 ng of Trypsin/LysC (Promega, Madison, Wisconsin, USA), on-bead overnight at room temperature. Peptides were then bound to an in-house-made C18 stage tip, washed with 0.1% formic acid, and stored at 4°C until LC-MS/MS analysis. After elution from the stage tips, samples were centrifuged in a SpeedVac to remove the acetonitrile. The peptide solution was then diluted with 0.1% formic acid before loading onto the column. Peptides were separated on a 30 cm pico-tip column (50 µm ID, New Objective/Ms Wil, Zurich, Switzerland) packed in-house with 3µm aquapur gold C18 material (Dr Maisch, Ammerbuch, Germany) using a 140- or 200-minute gradient (7% to 80% acetonitrile, 0.1% formic acid), delivered by an easy-nLC 1000 (Thermo Fisher Scientific), and electro-sprayed directly into an Orbitrap Fusion Tribrid Mass Spectrometer (Thermo Fisher Scientific). The data-dependent top-speed mode with a 1-second cycle time was used, in which a full scan over the 400–1500 mass range was performed at 240,000 resolution. The most intense ions (threshold of 5000 ions) were isolated by the quadrupole and fragmented with a higher-energy collisional dissociation collision of 30%. The maximum injection time of the ion trap was set at 35 milliseconds. The raw data matrix containing all proteins identified from immunoprecipitated cholangiocyte cell lysates with IgG4-RD or PSC IgG4 autoantibodies was imported into the proteomics analysis software Perseus (v1.5.5.3, MaxQuant, Max-Planck Institute of Biochemistry). Four technical replicates were used per patient sample. Data were log2-transformed and filtered for proteins only identified by site, reverse, or potential contaminants. In the obtained data matrix, proteins were annotated by NCBI gene names and full protein names. Log2 peptide intensities for annexin A11 (positive control), RPL22 (loading control), and prohibitins 1 and 2 were plotted for all individual patients. The mass spectrometry proteomics data have been deposited with the ProteomeXchange Consortium via the PRIDE partner repository with the dataset identifier PXD042856 (33).

### 2.5 Immunohistochemistry on human liver tissue

Sequentially sliced sections of paraffin-embedded human liver tissue were rehydrated by passing through xylene (three times, 7 min) followed by stepwise ethanol washing from 100% to 50%, each for 1 minute. After a wash in MQ, antigen retrieval was performed using 10 mM sodium citrate (Sigma Aldrich #C9999) at pH 6 at 98°C for 20 minutes. Subsequently, peroxidase blocking was performed using 0.3% hydrogen peroxide (VWR #8.22287.1000) for 30 minutes. Aspecific protein blocking was performed using Ultra V (Thermo Scientific #TA-125-UB). Incubation with primary antibodies against galectin-3 (Santa Cruz, raised in mouse), prohibitin 1 (Santa Cruz, raised in mouse), and cytokeratin 19 (Abcam, raised in rabbit) took place overnight at 4°C in a 1:100 dilution. The following day, the slides were washed three times in TBS. Slides were incubated with ready-to-use secondary horseradish peroxidase (HRP)-conjugated anti-rabbit/mouse IgG (BrightVision Immunologic #VWRKDPVO110HRP) for 1 hour at room temperature. The slides were washed three times in TBS. As a substrate, Vector NovaRed (HRP) (Vector Laboratories #SK-4800) was added to the slides for 40 min, after which the reaction was stopped in MQ. Counterstaining was performed by submerging the slides in hematoxylin for 2 min, after which the slides were washed in water for 10 min, followed by a single 3 min wash in MQ. Slides were briefly dried on a heater plate, after which they were mounted using Vectamount (Vector Laboratories #H-5000). Images were assessed and acquired on the Olympus BX51 brightfield microscope using the cellSens Entry software.

### 2.6 RNA-sequencing datasets

Count files of bulk RNA-sequenced human cholangiocyte models were obtained from Gene Expression Omnibus (GEO), GEO accession GSE156519, and our own dataset GSE221746, and imported into the R2 genomics analysis and visualization platform (http://r2.amc.nl/). Expression values were log2-transformed for each dataset and presented as individual heatmaps. Hepatocyte markers (*ALB* and *ASGR1*) and cholangiocyte markers (*EPCAM* and *KRT19*) were included as a phenotypic reference in the heatmaps.

### 2.7 Cell culture

H69 cholangiocytes were kindly provided by Dr. Douglas Jefferson (Tufts University, Boston, MS, USA). H69 cells were cultured in a 5% CO_2_ incubator as previously described and were passaged twice per week (34). The H69 cell culture medium composition can be found in **Supplementary Table S1**. Mycoplasma contamination tests were carried out every 3 months, with negative results throughout.

### 2.8 Lentivirus generation and transduction of shRNA-mediated knockdown

Short hairpin RNA (shRNA) constructs were purchased from the MISSION^®^ TRC version 1 shRNA library (Sigma-Aldrich, St. Louis, MO, USA): *LGALS3* (TRCN0000029305), *PHB1* (TRCN0000029207), *PHB2* (TRCN0000060922), and the non-targeting shRNA control (SHC002). The shRNAs against *LGALS3*, *PHB1*, and *PHB2* all targeted the coding sequence. Lentivirus was produced as previously described in an ML-2/BSL-2 facility (35). In brief, bacterial cultures were grown from which plasmid DNA was isolated using Midiprep kits (Genomed, LabNed). HEK 293T cells were plated in culture dishes and transfected the following day using polyethylenimine (PEI) with a mixture containing the plasmid of interest and the plasmids containing the lentiviral backbone. One day post-transfection, cell media was refreshed, and 72 hours after transfection, cell media containing lentivirus was harvested and stored appropriately. For the lentiviral transduction, H69 cholangiocytes were seeded in 6-well plates and transduced the following day using DEAE dextran and the harvested lentivirus. Six hours post-transduction, cell media was refreshed. After 1 day of recovery, the transduced H69 cholangiocytes were selected with 1 µg/ml puromycin to obtain stable *LGALS3*, *PHB1*, *PHB2*, and combined *PHB1/2* knockdown cholangiocytes.

### 2.9 RNA isolation, cDNA synthesis, and real-time quantitative PCR

H69 cholangiocytes were cultured in 12-well plates until confluency. Total RNA was isolated with a TRIzol reagent (Sigma #T9424). The RNA-containing phase was isolated with chloroform (Merck #2445) and centrifugation. RNA was recovered by precipitating with isopropyl alcohol (Merck #1040). Pellets were washed with 70% ethanol and resuspended in water treated with diethylpyrocarbonate (DEPC) (Sigma #D5757). Quality (A260/A280 > 1.8) and concentration of RNA samples were checked by spectrophotometry using the NanoDrop 1000 (Thermo Scientific, Waltham, MS, USA). Total RNA with 2 µg of input was treated with DNase I (Promega #M6101), followed by reverse transcription into cDNA using Random Hexamer primers (Promega #SO142), Oligo-dT primers (Invitrogen), and Revertaid transcriptase (Fermentas, #EP0442), resulting in a total volume of 20 µl cDNA. The cDNA was diluted to 100 μl, after which 2 μl of diluted cDNA served as a template for real-time quantitative PCR (RT-qPCR) with the SensiFAST SYBR No-ROX kit (Bioline). Primers were self-designed to cover all transcript variants of the gene of interest (**Supplementary Material** and **Supplementary Table S2** contain a list of methods and primer sequences). RT-qPCR plates were run on the Bio-Rad CXF96. Raw fluorescent values were exported, and starting concentration (N0) and cycle quantification (Cq) values were obtained using LinRegPCR (version 2013.0, Academic Medical Center, Amsterdam) (36). Expression levels were graphed relative to the geomean of human 36B4 (*RPLP0*) and *HPRT* reference genes, and normalized to the control condition.

### 2.10 Western blotting

Cells were lysed in radioimmunoprecipitation assay (RIPA) buffer, and lysate samples were prepared in Laemmli sample buffer. After running the samples on sodium dodecyl sulfate–polyacrylamide gel electrophoresis (SDS-PAGE) gels (10% Tris-Glycine), proteins were transferred by semi-dry blotting to polyvinylidene difluoride membranes (Millipore, Burlington, VT, USA), blocked for 2 hours at room temperature in 5% non-fat milk/TBST, and probed overnight at 4°C using the respective primary antibody in 5% non-fat milk/TBST (**Supplementary Table S3** for list of antibodies and dilutions). Immune complexes were detected using HRP-conjugated secondary antibodies and visualized using an enhanced chemiluminescence detection reagent (Lumi-light, Roche Diagnostics, Rotkreuz, Switzerland) and ImageQuant LAS 4000 (GE Healthcare, Chicago, IL, USA). Protein bands were quantified using ImageJ 1.50i (Wayne Rasband, National Institutes of Health, Bethesda, MD, USA). Results were presented as fold changes of sham (SHC002)-transduced H69 cells, normalized per experiment.

### 2.11 Bile acid permeation assay

Bile acid permeation assays were performed as previously described (22). H69 cholangiocytes were cultured until confluence in 24-well plates. Cells were refreshed with new culture medium 24 hours before the experiment. At the start of the experiment, cells were washed once with 500 µl of 20 mM HEPES-buffered Hank’s balanced salt solution (HBSS) pH 7.4 (Lonza #BE10-527F, Basel, Switzerland) and equilibrated for 30 minutes with HEPES-buffered HBSS pH 7.4 at 37°C in ambient air. HBSS was removed; 200 µl per well HEPES-buffered HBSS pH 7.4 with 2.87 kBq per well 22,23-^3^H-glycochenodeoxycholic acid (^3^H-GCDC), kindly provided by the late Dr. Alan Hofmann (University of California, San Diego, CA, USA), and 750 µM unlabeled GCDC (Sigma-Aldrich #G0759, St. Louis, MO, USA) were added. Excess bile acids were removed after 1, 4, 16, and 64 minutes to obtain kinetic readouts. Cells were washed once with ice-cold PBS and once with 200 µl of ice-cold PBS containing 2% fatty acid-free BSA (Sigma-Aldrich #A6003, St. Louis, MO, USA) to remove potentially membrane-bound GCDC. Cells were incubated with 50 µg/ml of digitonin (Merck, Kenilworth, NJ, USA) in ice-cold PBS to permeabilize the plasma membrane and extract the cytosolic fraction. Radioactivity in the cytosolic fraction was detected using the liquid scintillation counter Tri-carb 2900TR apparatus (PerkinElmer, Groningen, the Netherlands). Cells were then washed with ice-cold PBS and shaken in 0.05% SDS in MQ for 60 minutes on a plate shaker at room temperature. Bicinchoninic acid (BCA) assays were performed on both the cytosolic and membrane fractions to correct ^3^H-GCDC bile acid permeation values for total protein content per well. Experimental conditions tested in bile acid permeation experiments were as follows: GB1107, 10 µM, 24-hour pretreatment; recombinant galectin-3, 2.5 µg/ml, 24-hour pretreatment; rocaglamide, 100 nM, acute treatment; recombinant prohibitin 1, 0.25 µg/ml and 0.5 µg/ml, 24-hour pretreatment; patient-derived IgG preincubation, 50 µg IgG per 48 well, 48-hour pretreatment.

### 2.12 Caspase-3/7 assay

H69 cholangiocytes were plated in 96-well culture plates and grown until confluency. In parallel, an identical plate was seeded from the same cell suspensions and used for BCA assays to correct for potential differences in cell counts between shRNA control sham H69 cholangiocytes and *LGALS3*, *PHB1*, *PHB2*, and *PHB1/2* knockdown (KD) H69 cholangiocytes. The culture media were set to pH 6.9 and placed in a 5% CO_2_ incubator overnight. The following day, three conditions were set up, consisting of 1500 µM GCDC, 10 µM raptinal as a positive control of apoptosis induction, and an untreated negative control. pH was checked for all solutions and, where necessary, set back at pH 6.9. After the addition of the experimental solutions to the 96-well plate, cells were checked hourly for the initiation of apoptosis under a bright-field microscope. After 3–4 hours, 1500 µM GCDC with media set at pH 6.9 consistently induced rounding of the cells. For measuring Caspase-3/7 activity, the SensoLyte^®^ Homogeneous Rh110 Caspase-3/7 Assay Kit (AnaSpec #AS-71141) was used. Pre-warmed assay buffer at a volume of 5 ml was diluted with 5 ml of pre-warmed MQ and 200 µl of 0.5 M EDTA-NaOH pH 8.0, 200 µl of 1 M DTT, and, finally, 50 µl of (Z-DEVD)2-Rh110 as the fluorogenic indicator was added. Upon Caspase-3/7 cleavage, (Z-DEVD)2-Rh110 generates the Rh110 fluorophore, which was excited and detected at λEx = 496 nm/λEm = 520 nm on the CLARIOstar set at 37°C. Plates were read for 30 minutes. For analysis, slope values in the linear phase were obtained for each well. After assessing successful Caspase-3/7 induction by the positive control raptinal, Caspase-3/7 values were corrected for protein concentration and normalized to the untreated control sham condition. Experimental conditions tested in Caspase-3/7 experiments were as follows: GB1107, 10 µM; recombinant galectin-3, 2.5 and 5 µg/ml; rocaglamide, 100 nM; recombinant prohibitin 1, 0.25 µg/ml and 0.5 μg/ml. All treatments were added in the experimental solutions, i.e., GCDC, raptinal, or negative control.

### 2.13 Patient-derived IgG isolation

IgGs were isolated from human sera (healthy control, negative for anti-galectin-3 and anti-prohibitin 1 autoantibodies, and IRC patient #21, positive for anti-galectin-3 and anti-prohibitin 1 autoantibodies). In short, 350 µl of PureProteome beads (Millipore #LSKMAGAG10) were washed twice with wash buffer (PBS 0.01% Tween 20) and subsequently incubated with 350 µl of human serum in 1000 µl of wash buffer. Beads were then incubated for 30 minutes at room temperature, undergoing end-over-end mixing. Hereafter, beads were washed three times with wash buffer, and IgGs were subsequently eluted with 0.2 M glycine HCl pH 2.5 and neutralized with 1 M Tris pH 9. BCA assays were performed to determine the IgG content.

### 2.14 Statistical analysis

Experimental data are presented as means with standard deviations. Clinical data are represented as the median with an interquartile range. Statistical analyses were performed using GraphPad Prism 9 (GraphPad, La Jolla, CA, USA). The distribution of the data was assessed using the Kolmogorov–Smirnov test. For experimental data, the results of the two groups were compared with a paired or unpaired t-test where appropriate. A one-way ANOVA was used when comparing multiple groups. Outliers for RT-qPCR data were detected using the ROUT method. For clinical data, the Kruskal–Wallis test (for non-normally distributed data), one-way ANOVA (for normally distributed data), and the chi-square test (for categorical data) were used. Correlations were performed using Spearman’s rank correlation (for non-normally distributed data) or Pearson’s correlation (for normally distributed data). p-values <0.05 were considered statistically significant.

## 3 Results

### 3.1 Autoantibodies against galectin-3 and prohibitin 1 are present in a subset of people with IRC

**Figure 2:**
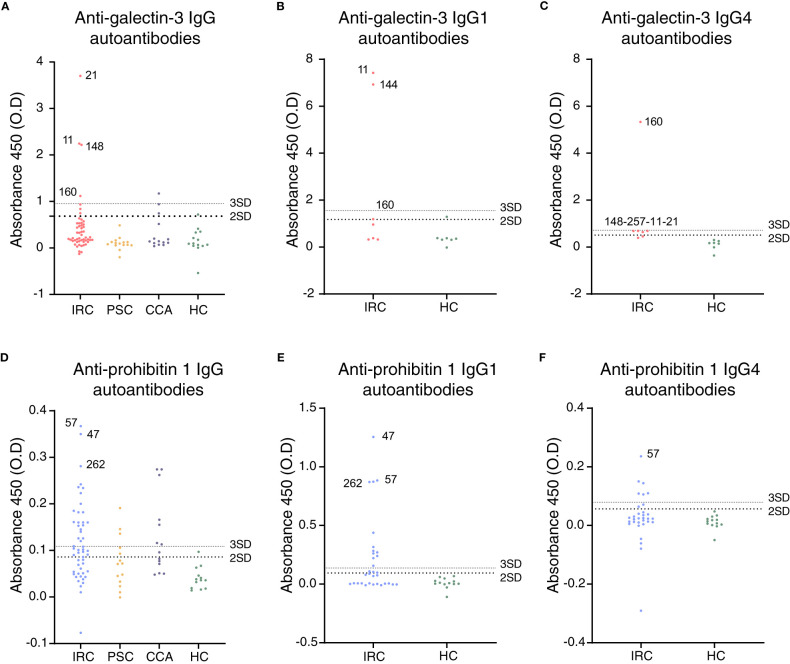
Galectin-3 and prohibitin 1 autoantibodies are present in a subset of people with IRC. **(A)** Anti-galectin-3 IgG, **(B)** IgG1, and **(C)** IgG4 autoantibody positivity in people with IRC, PSC, CCA, and healthy controls. Positive patients are annotated, and their further clinical details are presented in **Supplementary Table S4**. **(D)** Anti-prohibitin 1 IgG, **(E)** IgG1, and **(F)** IgG4 autoantibody positivity in people with IRC, PSC, CCA, and healthy controls. Further clinical details of positive patients are presented in **Supplementary Table S5**. **(A–F)** Data are represented as individual data points. Levels of significance: the cut-off for autoantibody positivity is defined as the mean plus two times the standard deviation of healthy controls (large dotted line); positivity based on the mean plus three times the standard deviation of healthy controls (small dotted line) is provided per patient in **Supplementary Tables S4, S5**. Autoantibody titer differences between groups. **(A)** HC *vs.* IRC, ns p = 0.1444; HC *vs.* PSC, ns p = 0.9879; HC *vs.* CCA, ns p = 0.6676; one-way ANOVA. **(B)** HC *vs.* IRC, ns p = 0.1161. **(C)** HC *vs.* IRC, ns p = 0.1124; unpaired t-tests. **(D)** HC *vs.* IRC, ** p = 0.0029; HC *vs.* PSC, ns p = 0.4550; HC *vs.* CCA, ** p = 0.0059; one-way ANOVA. **(E)** HC *vs.* IRC, * p = 0.0306. **(F)** HC *vs.* IRC, ns p = 0.4720; unpaired t-tests. CCA, cholangiocarcinoma; ELISA, enzyme-linked immunosorbent assay; HC, healthy control; IRC, IgG4-related cholangitis; PSC, primary sclerosing cholangitis; O.D., optical density.

**Figure 3:**
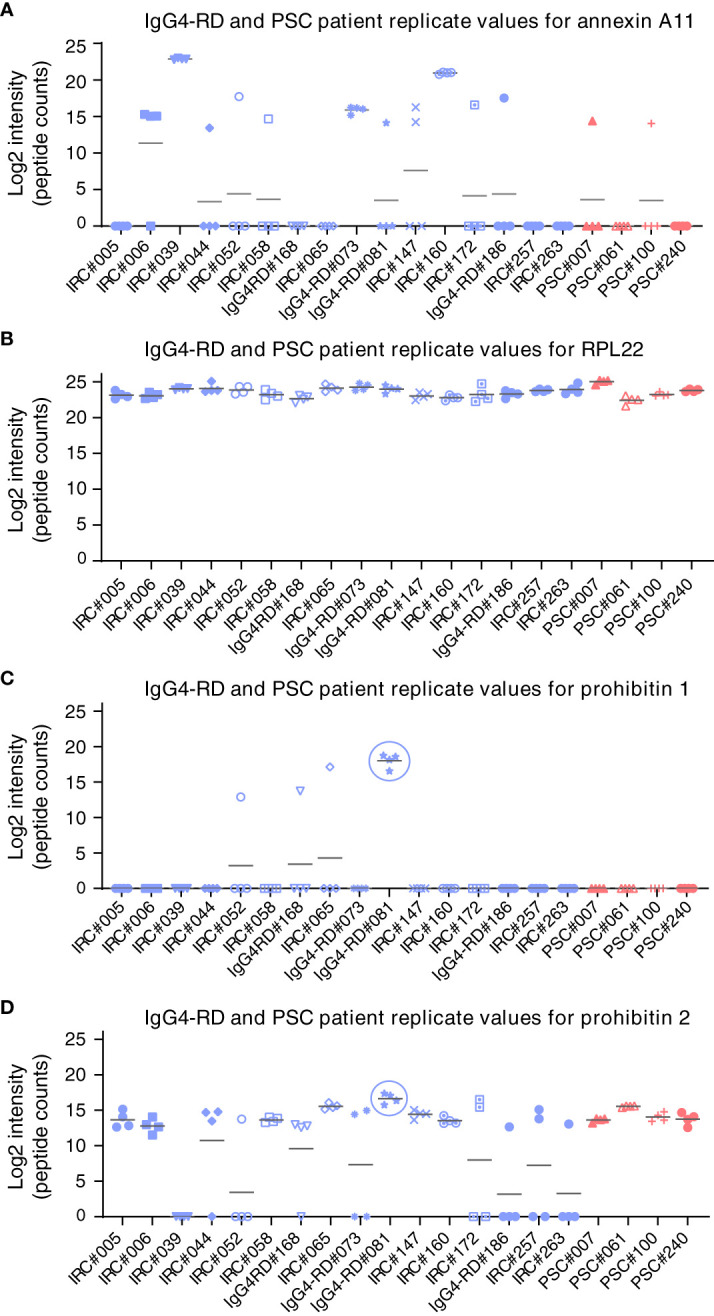
LC-MS/MS analysis of target antigens immunoprecipitated by patient-derived IgG4 autoantibodies. Label-free protein intensities (log2) were detected after immunoprecipitation by immobilized patient-derived IgG4 from human H69 cholangiocyte lysates. Four independent replicates were analyzed. **(A)** Annexin A11, **(B)** RPL22, **(C)** prohibitin 1, and **(D)** prohibitin 2. Gray bars represent the average values of four replicate measurements. IgG4-RD, IgG4-related disease; IRC, IgG4-related cholangitis; RPL22, ribosomal protein L22; PSC, primary sclerosing cholangitis; LC-MS/MS, liquid chromatography–tandem mass spectrometry.

First, autoantibody positivity directed against galectin-3 or prohibitin 1 was assessed in people with IRC (n = 52). Individuals with PSC (n = 14 and 15, resp.) and CCA (n = 14) were used as disease controls and healthy controls (n = 13 and 14, resp.). ELISAs for galectin-3 autoantibodies demonstrated that seven out of 52 people (13.5%) with IRC were positive, no people with PSC were positive, three out of 14 (21.4%) with CCA were positive, and one healthy control out of 14 (7.1%) was positive for galectin-3 autoantibodies, indicating that anti-galectin-3 autoantibodies are not specific for IRC (**Figure 2A**). Three of the people with IRC had specific IgG1 autoantibodies, and five had specific IgG4 autoantibodies directed against galectin-3 (**Figures 2B, C**). For prohibitin 1, 32 out of 52 people with IRC (61.5%) were positive for autoantibodies. In people with PSC, five out of 14 (35.7%) had autoantibodies against prohibitin 1, whereas, in people with CCA, eight out of 14 (57.1%) had autoantibodies against prohibitin 1, indicating that anti-prohibitin 1 autoantibodies are also not specific for IRC (**Figure 2D**). Furthermore, one healthy control was positive for anti-prohibitin 1 autoantibodies (7.1%). Fifteen of the people with IRC were positive for specific IgG1 autoantibodies, whereas eight were positive for specific IgG4 autoantibodies directed against prohibitin 1 (**Figures 2E, F**). Notably, neither IgG nor IgG1 or IgG4 autoantibody titers against galectin-3 or prohibitin 1 correlated with serum liver tests (total bilirubin, alkaline phosphatase (ALP), and gamma-glutamyl transferase (gGT)) when patients tested positive for autoantibodies (**Supplementary Figures S1, S2**). In a parallel approach, LC-MS/MS was performed on H69 cholangiocyte cell lysates after immunoprecipitation with patient-derived IgG4 coupled to sepharose beads. In line with previous findings, immunoprecipitation of annexin A11 was detected in three out of 16 individuals (19%) (**Figure 3**). An ELISA for anti-annexin A11 IgG4 autoantibodies detected annexin A11 in three out of 16 (19%) patients (**Supplementary Figure S3**). These results are similar to our previous findings and validate the LC-MS/MS approach. In this dataset, we did not detect peptides originating from galectin-3. Interestingly, one IgG4-RD patient (#081) with type 1 AIP demonstrated a pulldown of prohibitin 1.

Since prohibitin 1 and prohibitin 2 form a functional complex, we reasoned that prohibitin 2 could also be an autoimmune antigen. However, prohibitin 2 displays a detection pattern (although much less intense) that reflects background binding to the beads, as is illustrated by the ribosomal protein RPL22, which is present in all samples and serves as a loading control. This apparent aspecific binding makes it unfeasible to identify prohibitin 2 as an autoimmune antigen, although prohibitin 2 is detected at the highest intensity in the same patient (#81) who demonstrated prohibitin 1 pulldown, most likely indicating the pulldown of the functional prohibitin 1/2 complex. Thus, autoantibodies against galectin-3, but not against prohibitin 1, may be specific for a subgroup of people with IRC among those with fibrosing cholangiopathies, such as PSC.

People with IRC who were positive for anti-galectin-3 and anti-prohibitin 1 autoantibodies presented with (a tendency of) lower serum bilirubin, ALP, gGT, aspartate aminotransferase (AST), and alanine aminotransferase (ALT) levels (**Table 1**). Notably, people with IRC who were positive for anti-galectin-3 and anti-prohibitin 1 autoantibodies had high occurrences of major hepatopancreatobiliary (HPB) surgery in the past, possibly explaining in part the mild biochemical alterations after surgical removal of diseased tissue. People with anti-prohibitin 1 autoantibodies less frequently experienced multiorgan involvement. Other affected organs were most often secretory glands (**Supplementary Tables S4, S5**). Most patients were elderly men and had a history of blue-collar work, which we have previously identified as a risk factor for developing IRC (37). Four patients were positive for both galectin-3 and prohibitin 1 autoantibodies (**Supplementary Tables S4, S5**; patients #21, #160, #257, and #268). These patients did not have a more severe disease course (**Supplementary Figure S4**). In general, people with IRC were treated with standard immunosuppressive regimens with varying rates of clinical and biochemical responses (see **Supplementary Figure S4** for individual cases with high IgG1/IgG4 titer positivity and **Supplementary Tables S4, S5** for the complete overview). Thus, people with autoantibodies against galectin-3 or prohibitin 1 did not demonstrate a clinically more severe disease course compared to patients who were negative for these autoantibodies.

**Table 1:** Clinical differences between people with IRC positive and negative for anti-galectin-3 and prohibitin 1 autoantibodies.

	Galectin-3	Prohibitin 1	Negative	P value
Number of IRC patients	7 (13.5%)	32 (61.5%)	17 (32.7%)	
Sex (male subjects/female subjects)	7/0	28/4	14/2	0.6120
Age (years)	60 (48–72)	62 (51–67)	66 (60–71)	0.2258
Blue collar work (%)	5 (71.4%)	22 (68.8%)	12 (70.6%)	0.8974
Multiorgan(%)	6 (86%)	20 (62.5%)	15 (88.2%)	**0.0494**
Malignancy (%)	2 (29%)	13 (40.6%)	5 (29.4%)	0.7568
Major HPB surgery (%)	3 (75%)	14 (43.8%)	1 (5.9%)	**0.0233**
Bilirubin (µmol/L)	35 (18–74)	53 (17–122)	104 (41–187)	0.1464
ALP (IU/L)	232 (141–344)	294 (190–505)	402 (300–760)	**0.0436**
gGT (IU/L)	284 (101–1455)	423 (219–669)	621 (400–1026)	0.1318
AST (IU/L)	83 (36–144)	82 (40–132)	190 (72–306)	**0.0441**
ALT (IU/L)	117 (60–169)	110 (61–187)	350 (87–480)	0.0790
CA19.9 (kU/L)	60 (12–160)	135 (23–221)	412 (22–12836)	0.5651
CRP (mg/L)	1.1 (0.4–325)	23 (5.3–104)	28 (7.5–43)	0.5558
ESR (mm/hour)	30 (11.5–76)	48 (21–67)	65 (22–94)	0.5540

Data are presented as medians with an interquartile range. Normal distributed data were assessed by one-way ANOVA, and non-normal distributed data were assessed by the Kruskal–Wallis test.

Bold font indicates statistical significance (p<0.05).

### 3.2 Galectin-3, prohibitin 1, and prohibitin 2 are expressed in human cholangiocyte models

**Figure 4:**
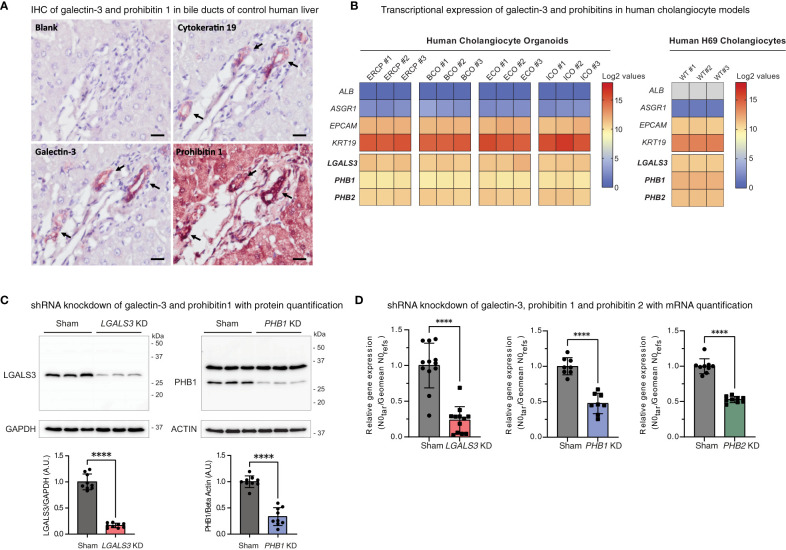
Galectin-3, prohibitin 1, and prohibitin 2 are abundantly expressed in human cholangiocyte models. **(A)** Immunohistochemical staining of galectin-3 and prohibitin 1 in control human liver sections with sequential cytokeratin 19 staining as a positive control for bile ducts (annotated by arrows) (scale bar, 20 µm). **(B)** Heatmaps depicting expression levels of galectin-3 and prohibitins 1 and 2 in human cholangiocyte models (positive for *EPCAM* and *KRT19* and negative for hepatocyte markers *ALB* and *ASGR1*). The left shows log2-transformed expression values of four different derivations of human cholangiocyte organoids (ERCP, BCO, ECO, and ICO). The right shows log2-transformed expression values of human H69 cholangiocytes. **(C)** LGALS3 (26 kDa) and PHB1 (30 kDa) expression and knockdown quantification in H69 sham transduced and respective shRNA knockdown cell lines. *LGALS3* KD is normalized by GAPDH (37 kDa) (9 cell samples from n = 3 independent experiments). *PHB1* KD is normalized by beta-actin (42 kDa) (9 cell samples from n = 3 independent experiments). Data are represented as means with standard deviations. **(D)** Relative mRNA expression of *LGALS3*, *PHB1*, and *PHB2* in H69 sham transduced and respective shRNA knockdown cell lines (9–12 cell samples from n = 3–4 independent experiments, 1 outlier value excluded from the sham and *PHB1* dataset). Data are represented as the starting concentration (N0) of target genes over the geomean of the reference genes *36B4* (*RPLP0*) and *HPRT* N0 values normalized to sham. Levels of significance: **(C)** *LGALS3* KD **** p < 0.0001 and *PHB1* KD **** p < 0.0001, unpaired t-tests. **(D)** *LGALS3* KD **** p < 0.0001, *PHB1* KD **** p < 0.0001, *PHB2* KD **** p < 0.0001, unpaired t-tests. *ALB*, albumin; A.U, arbitrary units; *ASGR1*, asialoglycoprotein receptor 1; BCO, bile-collected organoid (from resected gallbladders and PTC drainage); ECO, extrahepatic cholangiocyte organoid; *EPCAM*, epithelial cell adhesion molecule; ERCP, ERCP-derived cholangiocyte organoid; *GAPDH*, glyceraldehyde-3-phosphate dehydrogenase; ICO, intrahepatic cholangiocyte organoid; IHC, immunohistochemistry; kDa, kilodalton; KD, knockdown; *KRT19*, cytokeratin 19; *LGALS3*, galectin-3; N0, starting concentration; *PHB1*, prohibitin 1; *PHB2*, prohibitin 2; WT, wild type.

As galectin-3 and prohibitin 1 autoantibodies can be detected in people with IRC, we assessed the cholangiocellular expression of these proteins in control human liver tissue by immunohistochemistry. Galectin-3 staining resulted in a cholangiocellular signal in the bile ducts, and prohibitin 1 staining demonstrated positivity in both cholangiocytes and hepatocytes (**Figure 4A**). Furthermore, the expression of galectin-3 and prohibitins 1 and 2 was assessed in various human cholangiocyte models at the transcriptional level (**Figure 4B**). To this end, a publicly available bulk RNA-sequencing dataset on four types of human cholangiocyte organoids was assessed (GEO: GSE156519). In addition, RNA sequencing on human H69 cholangiocytes was performed (GEO: GSE221746). A distinct cholangiocyte phenotype was demonstrated in all cholangiocyte organoids and human H69 cholangiocytes by high expression of the cholangiocellular markers *EPCAM* and *KRT19*, while the expression of hepatocyte markers *ALB* and *ASGR1* was low. Expression at the mRNA level of *LGALS3*, *PHB1*, and *PHB2* was evident across all types of cholangiocyte organoids (endoscopic retrograde cholangiopancreatography (ERCP)-derived, bile-collected, extrahepatic, and intrahepatic) and in human H69 cholangiocytes (**Figure 4B**). Thus, galectin-3 and prohibitins 1 and 2 are abundantly expressed in cholangiocytes, the major cell type affected in IRC.

To investigate the cellular roles of galectin-3, prohibitin 1, and prohibitin 2 in cholangiocytes, stable knockdown cell lines were generated (**Figures 4C, D**). In addition to single gene knockdown cell lines, as prohibitin 1 and prohibitin 2 form a multimeric complex and can likely be immunoprecipitated as a complex by patient-derived IgG4, both proteins were simultaneously knocked down in a combined *PHB1/2* knockdown cell line. Knockdown efficiency was assessed by Western blotting and qPCR analysis (*LGALS3* 83% KD on Western blotting (WB), 76% KD on RT-qPCR; *PHB1* 67% KD on WB, 52% KD on RT-qPCR; *PHB2* 47% KD on RT-qPCR; **Figures 4C, D**).

### 3.3 Galectin-3 knockdown, pharmacological inhibition, and recombinant protein substitution do not demonstrate a clear-cut protective effect against toxic bile acids in human cholangiocytes

**Figure 5:**
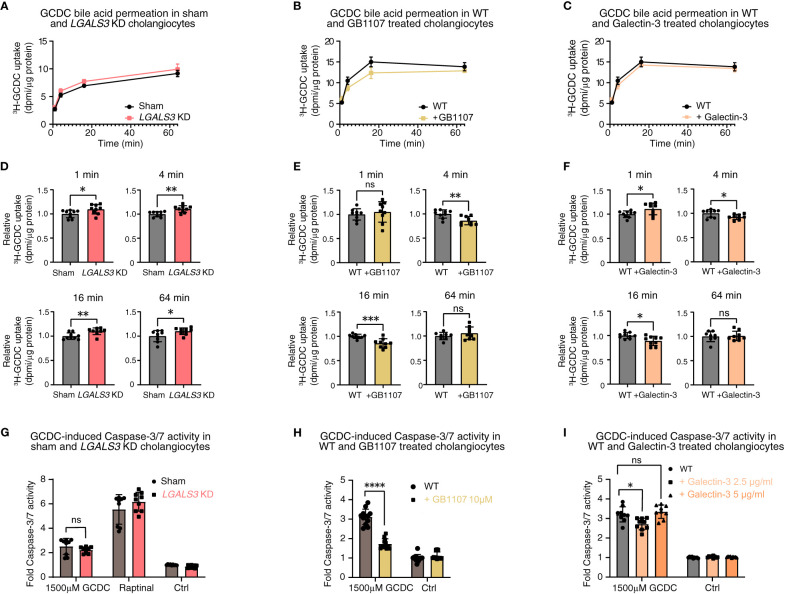
Galectin-3 knockdown, pharmacological inhibition, and recombinant protein substitution do not demonstrate a clear damaging or protective effect against toxic bile acids in human cholangiocytes. 22,23-^3^H-GCDC permeation assay in **(A)** sham and *LGALS3* KD cholangiocytes, **(B)** WT and GB1107-treated cholangiocytes, and **(C)** WT and recombinant galectin-3-treated cholangiocytes (representative experiments of n = 3). Relative quantification of 22,23-^3^H-GCDC permeation per time point in **(D)** sham and *LGALS3* KD cholangiocytes, **(E)** WT and GB1107-treated cholangiocytes, and **(F)** WT and recombinant galectin-3-treated cholangiocytes (8–9 samples from n = 3 independent experiments). GCDC-induced Caspase-3/7 activity in **(G)** sham and *LGALS3* KD cholangiocytes, **(H)** WT and GB1107-treated cholangiocytes, and **(I)** WT and recombinant galectin-3 treated cholangiocytes (9–12 samples from n = 3–4 independent experiments). Data are represented as means with standard deviations. Levels of significance: **(D)** 1 min: * p = 0.0353. 4 min: ** p = 0.0023. 16 min: ** p = 0.0070. 64 min: * p = 0.0365, unpaired t-tests. **(E)** 1 min: ns, p = 0.5371. 4 min: ** p = 0.0034. 16 min: *** p = 0.0007. 64 min: ns, p = 0.2797, unpaired t-tests. **(F)** 1 min: * p = 0.0311. 4 min: * p = 0.0321. 16 min: * p = 0.0104. 64 min: ns, p = 0.8328, unpaired t-tests. **(G)** ns, p = 0.2204, unpaired t-test. **(H)** **** p < 0.0001, unpaired t-test. **(I)** In order of sham to 2.5 µg/ml, to 5 µg/ml: * p = 0.0103, ns, p = 0.6212, one-way ANOVA. Ctrl, control; DPMI, disintegrations per minute; GCDC, glycochenodeoxycholic acid; KD, knockdown; *LGALS3*, galectin-3; ns, not significant; WT, wild type.

Based on the role of galectin-3 in the apical sorting of membrane proteins, we hypothesized that galectin-3 could exert a protective effect against toxic bile acids in human cholangiocytes by strengthening the biliary bicarbonate umbrella. Therefore, we used three parallel *in vitro* approaches, namely, stable *LGALS3* knockdown, galectin-3 inhibition by GB1107, and recombinant galectin-3 treatment. We assessed the effects of these parallel approaches by three assays that touch on the stability of the biliary bicarbonate umbrella: i) intracellular pH (pH_i_) measurements using BCECF AM; ii) radioactive bile acid influx by measuring 22,23-^3^H-glycochenodeoxycholic acid (^3^H-GCDC) permeation; and iii) GCDC-induced apoptosis determined by Caspase-3/7 assays (22, 24). Neither stable knockdown of *LGALS3* nor galectin-3 inhibition with GB1107 induced alterations in pH_i_, whereas recombinant galectin-3 treatment lowered the intracellular pH of human cholangiocytes (**Supplementary Figure S5**). To assess the role of galectin-3 in protecting cholangiocytes against toxic bile acids, we performed radioactive bile acid permeation assays. Stable *LGALS3* knockdown led to slightly increased toxic bile acid permeation after 1, 4, 16, and 64 minutes. In contrast, its acute counterpart—GB1107 treatment—conflictingly lowered toxic bile acid permeation at 4 and 16 minutes. Treatment with recombinant galectin-3 led to a slightly decreased toxic bile acid permeation at 4 and 16 minutes (**Figures 5A–F**). An increased rate of toxic bile acid permeation into cholangiocytes is expected to eventually induce cholangiocyte apoptosis. To assess this final downstream pathway, we performed Caspase-3/7 assays after exposure to GCDC. Stable *LGALS3* knockdown did not result in an increased rate of apoptosis, despite the increased toxic bile acid permeation. Acute inhibition of galectin-3 by GB1107 strongly reduced GCDC-induced apoptosis. Nevertheless, recombinant galectin-3 treatment only reduced apoptosis at a lower dose of 2.5 μg/ml, but not at a higher dose of 5 μg/ml (**Figures 5G–I**). Thus, our data do not indicate a clear role for galectin-3 in cholangiocyte protection against toxic bile acids under the experimental conditions chosen.

### 3.4 Prohibitin knockdown, pharmacological inhibition and recombinant protein substitution do not disclose a role of prohibitins in the human cholangiocyte defense against toxic bile acids

**Figure 6:**
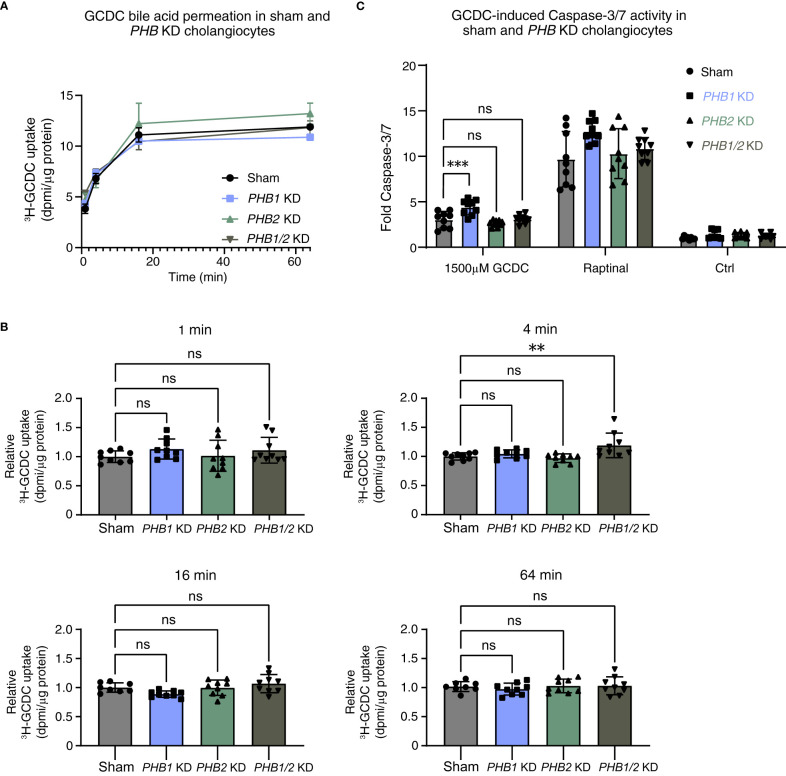
Single lentiviral shRNA knockdown of *PHB1* or *PHB2* or double *PHB1/2* knockdown demonstrates a clear damaging effect against toxic bile acids in human cholangiocytes. 22,23-^3^H-GCDC permeation assay in **(A)** sham, *PHB1* KD, *PHB2* KD, and combined *PHB1/2* KD cholangiocytes (representative experiments of n = 3). Relative quantification of 22,23-^3^H-GCDC permeation per time point in **(B)** sham, *PHB1* KD, *PHB2* KD, and combined *PHB1/2* KD cholangiocytes (8–9 samples from n = 3 independent experiments). GCDC-induced Caspase-3/7 activity in **(C)** sham, *PHB1* KD, *PHB2* KD, and combined *PHB1/2* KD cholangiocytes (9–12 samples from n = 3–4 independent experiments). Data are represented as means with standard deviations. Levels of significance: **(B)** in order of sham to *PHB1* KD, to *PHB2* KD, to *PHB1/2* KD: 1 min: ns, p = 0.3731, ns, p = 0.9966, ns, p = 0.5143. 4 min: ns, p = 0.7795, ns, p = 0.9155, ** p = 0.0060. 16 min: ns, p = 0.1287, ns, p = 0.9999, ns, p = 0.4846. 64 min: ns, p = 0.7828, ns, p = 0.9940, ns, p = 0.9902, one-way ANOVA. **(C)** In order of sham to *PHB1* KD, to *PHB2* KD, to *PHB1/2* KD: *** p = 0.0005, ns, p = 0.4392, ns, p = 0.9985, one-way ANOVA. Ctrl, control; DPMI, disintegrations per minute; GCDC, glycochenodeoxycholic acid; KD, knockdown; ns, not significant; *PHB1*, prohibitin 1; *PHB2*, prohibitin 2; WT, wild type.

We hypothesized that prohibitins—in analogy with the IRC autoantigens annexin A11 and laminin 511-E8 and consideration of a potential pathogenetic role of prohibitin 1 in PBC (30)—could contribute to the protection of human cholangiocytes against toxic bile acid influx and stabilization of the biliary bicarbonate umbrella. As for galectin-3 (see above), we used three parallel *in vitro* approaches: i) stable *PHB1* or *PHB2* knockdown and combined *PHB1/2* knockdown; ii) pan-prohibitin inhibition by rocaglamide; and iii) recombinant prohibitin 1 treatment. We assessed the effects of these parallel approaches using the three above-described assays that touch on the stability of the biliary bicarbonate umbrella. Stable knockdown of *PHB1* and combined knockdown of *PHB1/2* showed a decrease in the pH_i_ of human cholangiocytes (**Supplementary Figure S6**). To assess whether prohibitins protect cholangiocytes against toxic bile acid influx, we performed radioactive bile acid permeation assays. Under stable knockdown conditions, only the combined *PHB1/2* knockdown showed a slight increase in toxic bile acid permeation after 4 minutes, but not at other time points (**Figures 6A, B**). Assessment of the final downstream apoptotic pathway showed a slight increase in GCDC-induced apoptosis in the *PHB1* knockdown cell line (**Figure 6C**).

**Figure 7:**
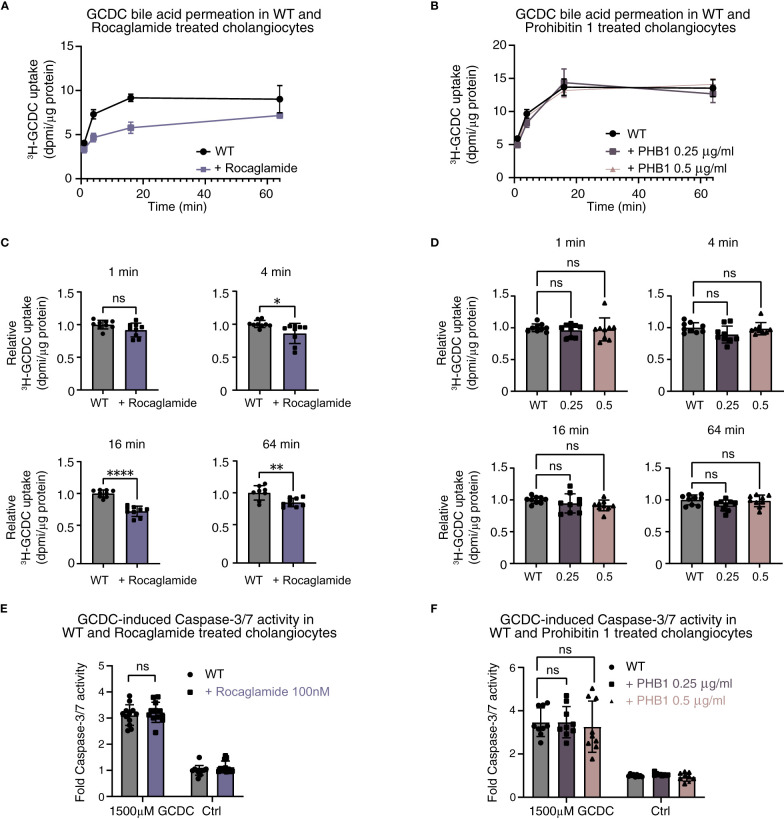
Pharmacological inhibition of prohibitins and recombinant protein substitution of prohibitin 1 do not demonstrate a clear damaging or protective effect against toxic bile acids in human cholangiocytes. 22,23-^3^H-GCDC permeation assay in **(A)** WT and rocaglamide-treated cholangiocytes; **(B)** WT, recombinant low-dose prohibitin 1 0.25 µg/ml, and recombinant high-dose 0.5 µg/ml-treated cholangiocytes (representative experiments of n = 3). Relative quantification of 22,23-^3^H-GCDC permeation per time point in **(C)** WT and rocaglamide-treated cholangiocytes, and **(D)** WT, recombinant low-dose prohibitin 1 0.25 µg/ml and recombinant high-dose 0.5 µg/ml-treated cholangiocytes (8–9 samples from n = 3 independent experiments). GCDC-induced Caspase-3/7 activity in **(E)** WT and rocaglamide-treated cholangiocytes, and **(F)** WT, recombinant low-dose prohibitin 1 0.25 µg/ml and recombinant high-dose 0.5 µg/ml-treated cholangiocytes (9–12 samples from n = 3–4 independent experiments). Data are represented as means with standard deviations. Levels of significance: **(C)** 1 min: ns, not significant p = 0.0634. 4 min: * p = 0.0167. 16 min: **** p < 0.0001. 64 min: ** p = 0.0041, unpaired t-tests. **(D)** In order of sham to 0.25 µg/ml, to 0.5 µg/ml: 1 min: ns, p = 0.7354, ns, p = 0.8690. 4 min: ns, p = 0.0737, ns, p = 0.9288. 16 min: ns, p = 0.4439, ns, p = 0.1545. 64 min: ns, p = 0.0791, ns, p = 0.9104, one-way ANOVA. **(E)** ns, p = 0.5108, unpaired t-test. **(F)** In order of sham to 0.25 µg/ml, to 0.5 µg/ml: ns, p > 0.9999, ns, p = 0.8369, one-way ANOVA. Ctrl, control; DPMI, disintegrations per minute; GCDC, glycochenodeoxycholic acid; KD, knockdown; ns, not significant; PHB1, prohibitin 1; WT, wild type.

In line with results after the stable knockdown of *PHB1* and combined knockdown of *PHB1/2*, the pan-prohibitin inhibitor rocaglamide induced a decrease in pH_i_ in human cholangiocytes (**Supplementary Figure S7**) and markedly reduced toxic bile acid influx (**Figures 7A, C**). However, treatment with recombinant prohibitin 1 conflictingly also showed a decrease in pH_i_ (**Supplementary Figure S7**) but did not affect toxic bile acid influx in human cholangiocytes (**Figures 7B, D**) or GCDC-induced apoptosis (**Figures 7E, F**). Thus, our data do not show evidence for a potentially relevant protective effect of prohibitins against human toxic bile acids in human cholangiocytes *in vitro*.

### 3.5 Pretreatment of cholangiocytes with patient-derived IgG positive for anti-galectin-3 and anti-prohibitin 1 autoantibodies does not lead to increased toxic bile acid permeation

**Figure 8:**
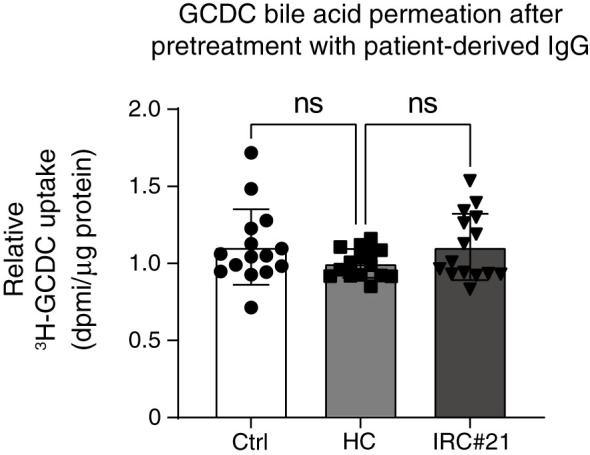
Pretreatment of cholangiocytes with patient-derived IgG positive for anti-galectin-3 and anti-prohibitin 1 autoantibodies does not lead to increased toxic bile acid permeation. Relative quantification of 22,23-^3^H-GCDC permeation into vehicle control cholangiocytes (Ctrl, white), cholangiocytes treated with IgG from a healthy control negative for anti-galectin-3 and anti-prohibitin 1 autoantibodies (HC, light gray), cholangiocytes treated with IgGs isolated from an IRC patient (#21, dark gray), and positive for anti-galectin-3 and anti-prohibitin 1 autoantibodies. The time point is 16 min incubation with 22,23-^3^H-GCDC (15 samples from n = 5 independent experiments). Levels of significance: HC vs Ctrl ns, p = 0.2474. HC vs IRC#21 ns, p = 0.2410. One-way ANOVA. Ctrl, vehicle control; HC, healthy control; IRC#21, IgG4-related cholangitis patient #21.

As a final experiment, IgGs were isolated from one healthy control (negative for anti-galectin-3 and anti-prohibitin 1 autoantibodies) and one IRC patient (#21) who was positive for anti-galectin-3 and anti-prohibitin 1 autoantibodies (see **Figure 2A** and **Supplementary Tables S4, 5**). Pretreatment with patient-derived IgG for 48 hours did not lead to increased GCDC-influx after 16 minutes of incubation (**Figure 8**).

## 4 Discussion

The current study is the first to demonstrate that a subset of people with IRC form autoantibodies directed against galectin-3 or prohibitin 1. Autoantibodies against galectin-3 were detected in people with IRC (13.5%) and CCA (21.4%) but not in people with PSC and were of both the IgG1 and IgG4 subclasses. Prohibitin 1 autoantibodies occurred frequently (61.5%) in people with IRC but were also frequently detected in people with PSC (35.7%) and were partly of the IgG1 or IgG4 subclass. Neither IgG nor IgG1 or IgG4 titers against galectin-3 or prohibitin 1 correlated with patients’ serum liver parameters.

Furthermore, the potential role of galectin-3 and prohibitins 1 and 2 in protecting cholangiocytes against toxic bile acids as previously reported by us for the IRC autoantigens annexin A11 (22) and laminin 511-E8 (25) was assessed in the current study *in vitro* by parallel approaches of stable gene knockdown, pharmacological inhibition, and recombinant galectin-3 or prohibitin 1 treatment. These parallel approaches did not demonstrate clear-cut protective effects in three assays that touch on the functionality of the biliary bicarbonate umbrella (23, 24), including intracellular pH measurements, toxic bile acid permeation studies, and experiments determining GCDC-induced cholangiocyte apoptosis. In line with these findings, pretreatment with IgGs isolated from an IRC patient with anti-galectin-3 and anti-prohibitin 1 autoantibodies did not lead to increased GCDC permeation into cholangiocytes.

Galectin-3 and prohibitin 1 autoantibody positivity have previously been assessed in more clinically diverse cohorts of people with IgG4-RD who had multiorgan manifestations. Galectin-3 and prohibitin 1 autoantibodies were detected at higher rates (28% and 73.5%, respectively) than in our IRC-specific cohort (11, 13). This difference in autoantibody positivity could potentially arise from differences in the genetic backgrounds of the cohorts, differences in toxin exposure (37), or differences in disease severity. It should be noted that the recombinant galectin-3 and prohibitin 1 proteins used in these studies and our ELISA experiments were produced in *Escherichia coli*, which lack post-translational modifications such as glycosylation (38). This could potentially lead to false-positive or false-negative results, as glycosylation could affect (auto)antibody binding (39).

As an alternative approach to detecting autoantibodies against galectin-3 and prohibitin 1, we made use of a limited cohort of people with IgG4-RD, where LC-MS/MS was performed after patient-derived IgG4 had been immobilized to immunoprecipitate antigenic targets from H69 cholangiocyte lysates. In this dataset, galectin-3 was not detected, in contrast to the identification of five IgG4 anti-galectin-3-positive patients detected by our ELISA. This difference could potentially be explained by the different techniques (ELISA *vs.* LC-MS/MS), as this has previously been shown to affect antigen detection in IgG4-RD (12), by the sensitivity of the LC-MS/MS, the small group size of the LC-MS/MS cohort, or the fact that only one positive patient (#160) was analyzed in both cohorts. Patient-derived IgG4 beads pulled down prohibitin 1 in one patient with type 1 AIP but not in patients with IRC, which is in line with our ELISA data demonstrating scarce and low titer positivity of IgG4 subclass autoantibodies against prohibitin 1 in people with IRC. In the same patient with type 1 AIP where prohibitin 1 was detected, prohibitin 2 could reliably be detected by MS2 identification, most likely as a consequence of the pulldown of the functional prohibitin 1/2 complex. Whether prohibitin 2 can be a genuine autoantigen remains to be resolved, as prohibitin 2 displays aspecific binding in our assay. We validated our LC-MS/MS approach by performing an ELISA for the previously identified autoantigen annexin A11 (10). It is noteworthy that two IRC patients were assessed positively by both methods: one patient was only detected by LC-MS/MS, and one patient was only detected by ELISA. The diverging results when applying these two methods could potentially arise from false-positive or false-negative findings in our ELISA, as our approach with recombinant proteins derived from *Escherichia coli* could affect autoantibody binding.

Notably, in the clinically diverse galectin-3 IgG4-RD cohort, only 15% of the people who were positive for galectin-3 autoantibodies had a history of IRC, indicating that galectin-3 positivity in IgG4-RD does not de facto lead to the development of IRC. The frequent detection of prohibitin 1 autoantibodies in people with PSC and CCA indicates that anti-prohibitin 1 autoantibodies are aspecific and occur in other immune-mediated cholestatic liver diseases. Although complicated by retrospective analysis, our other clinical data question the direct role of galectin-3 and prohibitin 1 autoantibodies in affecting cholangiocyte defense against toxic bile acids, as i) autoantibody titers against galectin-3 and prohibitin 1 do not correlate with cholestatic serum markers, and ii) patients positive for galectin-3 and prohibitin 1 autoantibodies had (a tendency of) lower cholestatic serum markers compared to patients without galectin-3 and prohibitin 1 autoantibodies. If galectin-3 and prohibitins were to protect cholangiocytes against bile acid toxicity, direct inhibition of their endogenous function by autoantibodies would most likely result in higher cholestatic serum markers, and positive correlations between autoantibody titers and cholestatic serum markers would be expected. Indeed, pretreatment with IgGs isolated from a patient with anti-galectin-3 and anti-prohibitin-1 autoantibodies did not lead to an increase in toxic bile acid permeation into cholangiocytes.

Our *in vitro* experiments, which assess the function of galectin-3 through parallel approaches (stable *LGALS3* knockdown, galectin-3 inhibition with GB1107, and recombinant galectin-3 treatment) have to be interpreted in light of the ubiquitous roles of galectin-3, which strongly depend on its localization (extracellular versus intracellular) (26). Galectin-3 is secreted from epithelial cells to subsequently undergo endosomal uptake and regulate the apical sorting of membrane proteins (27, 28, 40–43). As a robust expression of ion transporters on the apical cholangiocyte membrane is essential for the formation of a protective biliary bicarbonate umbrella (22–24), we investigated the possibility that galectin-3 could be involved in the apical sorting of membrane proteins that stabilize the biliary bicarbonate umbrella. As the transporters involved in stabilizing the biliary bicarbonate umbrella regulate the secretion of bicarbonate and hydrogen ions, intracellular pH measurements were performed after stable *LGALS3* knockdown, galectin-3 inhibition by the membrane permeable inhibitor GB1107, and recombinant galectin-3 treatment. Neither *LGALS3* knockdown nor GB1107 treatment affected the intracellular pH of human cholangiocytes. Although recombinant galectin-3 treatment did result in a decreased intracellular pH, this was not accompanied by a clear-cut reduction in toxic bile acid permeation after recombinant galectin-3 treatment. Additionally, if galectin-3 were to stabilize the biliary bicarbonate umbrella, *LGALS3* knockdown and GB1107 treatment would be expected to lead to increased toxic bile acid permeation. However, stable *LGALS3* knockdown only slightly increased toxic bile acid permeation, whereas GB1107 treatment conversely reduced toxic bile acid permeation after early time points. These findings indicate that galectin-3 is most likely not involved in the regulation of cholangiocellular bicarbonate secretion, which is in line with the finding that galectin-3 knockout mice only demonstrate increased chloride excretion but no changes in bicarbonate secretion (44).

The interpretation of the results on GCDC-induced apoptosis is complicated by the fact that both pro- and anti-apoptotic roles have been demonstrated for galectin-3. Whether galectin-3 exerts a pro- or anti-apoptotic effect is mostly dependent on the cellular localization of galectin-3, with anti-apoptotic effects described for intracellular galectin-3 and pro-apoptotic effects demonstrated for extracellular galectin-3. Although the role of extracellular galectin-3 has been described as pro-apoptotic (45), overexpression of galectin-3 has been shown to increase cell adhesion to the extracellular matrix, thereby conferring resistance to various apoptotic stimuli (46). Of particular interest here is the role of galectin-3 in promoting cell adhesion to laminin 511, another autoantigen in IRC for which a protective role in cholangiocytes against toxic bile acids has been described (25, 47). These opposing mechanisms of extracellular galectin-3 and the balance with its anti-apoptotic intracellular counterpart could potentially explain the diverging results seen in GCDC-induced apoptosis after *LGALS3* knockdown, galectin-3 inhibition with GB1107, and recombinant galectin-3 treatment in varying doses.

Similar to our studies on the role of galectin-3 in cholangiocytes, three approaches were chosen to assess the role of prohibitins 1 and 2, including stable *PHB1*, *PHB2*, and combined *PHB1/2* knockdown, pan-prohibitin inhibition by rocaglamide, and recombinant prohibitin 1 treatment. Remarkably, all interventions except *PHB2* knockdown led to a decrease in intracellular pH. Although hard evidence is lacking, prohibitins and proteins with prohibitin domains have been suggested to regulate the activity of ion channels by altering the local lipid environment in the plasma membrane (48, 49). Alternatively, prohibitin 1 is a known binding partner to the ion channel regulator annexin A2 (50). This decrease in intracellular pH did not translate into an attenuated toxic bile acid permeation except after rocaglamide treatment. Rocaglamide is a natural prohibitin 1 and 2 ligand that blocks the downstream effects of these proteins. However, as rocaglamide’s mechanisms of action are vast and also include translational inhibition (51, 52), the experiments performed in human H69 cholangiocytes exposed to rocaglamide must be interpreted with caution. Just like galectin-3, prohibitins 1 and 2 also have a complex role in apoptosis, and their pro- or anti-apoptotic effects depend on the cell type, their subcellular localization, and post-translational modifications (53). In our experiments, only isolated *PHB1* knockdown led to an increased rate of GCDC-induced apoptosis.

Although our experimental and clinical data do not point to a role for galectin-3 and prohibitins in protecting cholangiocytes against toxic bile acids, galectin-3 and prohibitins could still be involved in the pathogenesis of IRC via other mechanisms. IRC is characterized by aberrant B- and T-cell responses, which can be observed as a lymphoplasmacytic tissue infiltrate and lead to the formation of storiform fibrosis in and around the bile ducts. Notably, galectin-3 plays an important role in regulating B- and T-cell function. Galectin-3 has been shown to inhibit the differentiation of B cells into plasma cells, and galectin-3 knockout mice show increased numbers of plasma cells and develop hypergammaglobulinemia upon infection (54, 55). In addition, spontaneous anti-laminin-secreting plasma cells have been detected in galectin-3 knockout mice (56). These findings are of interest in the context of IgG4-RD’s pathogenesis, as affinity-matured B-cell receptor clones have been identified by us and others in people with IgG4-RD (7–9). With regard to T cells, galectin-3 has been shown to induce T-cell apoptosis (57, 58) and negatively regulate TCR-mediated CD4^+^ T-cell activation (59). Furthermore, galectin-3 plays a relevant role in the formation of (liver) fibrosis (60, 61), and specific galectin-3 inhibitors have been developed to target this process (62).

Also, prohibitins play an apparent role in the regulation of B- and T-cell function and the formation of fibrosis. In B cells, prohibitins 1 and 2 regulate IgG1 production, which is of interest as people with IRC frequently have targeted autoantibodies of the IgG1 subclasses (63). In activated T cells, prohibitin 1 and prohibitin 2 are upregulated (64), with proteomics data showing that prohibitins are particularly expressed on the cell surface of human Th17 cells, where they maintain the stability of Th17 cells (65). This is relevant as IgG4-RD, but other immune-mediated cholestatic liver diseases, such as PSC, are associated with an increased Th17 response (66, 67). Furthermore, prohibitin 1 plays an important role in the formation of fibrosis, as liver-specific *Phb1^−/−^* mice spontaneously develop liver fibrosis (68).

Collectively, as we have recently demonstrated that the IRC autoantigens annexin A11 and laminin 511-E8 both display a protective role in human cholangiocytes against toxic bile acids, we hypothesized a similar common role for galectin-3 and prohibitins. Despite the literature supporting this hypothesis and our clinical data demonstrating that galectin-3 and prohibitin 1 are autoantigens in people with IRC, our experimental *in vitro* data did not demonstrate a clear-cut protective role of galectin-3 and prohibitins in human cholangiocytes against toxic bile acids. In addition, pretreatment with autoantibodies directed against galectin-3 and prohibitin 1 did not lead to increased toxic bile acid permeation into cholangiocytes. It is, therefore, unlikely that autoantibodies directed against galectin-3 and prohibitin 1 would directly lead to cholangiocyte damage by interfering with the cholangiocellular functions of galectin-3 and prohibitins. However, as both galectin-3 and prohibitins are involved in pathophysiological processes that are implicated in IRC, such as regulation of B- and T-cell function and the formation of fibrosis, direct autoantibody-induced functional impairment of galectin-3 and prohibitin 1 could still play a role in the pathogenesis of IRC. Alternatively, autoantibody binding to galectin-3 or prohibitin 1 could elicit an excessive immune response that could lead to the obstructive cholestasis seen in IRC. Thus, our studies on the role of the autoantigens galectin-3 and prohibitin 1 in IRC did not clearly disclose a protective function of these autoantigens in human cholangiocytes against toxic bile acids.

## Data availability statement

The mass spectrometry proteomics data have been deposited with the ProteomeXchange Consortium via the PRIDE partner repository with the dataset identifier PXD042856. The RNA sequencing dataset of human H69 cholangiocytes used for this study has been deposited in the R2 genomics analysis and visualization platform and the Gene Expression Omnibus (GEO) and can be openly accessed under GEO accession GSE221746.

## Ethics statement

The studies involving humans were approved by the Medical Ethics Committee of Amsterdam University Medical Centers (formerly Academic Medical Center Amsterdam). The studies were conducted in accordance with local legislation and institutional requirements. The participants provided their written informed consent to participate in this study.

## Author contributions

RK, DCT, SG, and UB conceived the experiments. RK, DCT, and DT performed the experiments. RK, DCT, DT, HV, SG, and UB analyzed the data. The original draft of the manuscript was written by RK, DCT, and UB, after which reviewing and editing was done by all authors. The final manuscript was approved by all authors.

## Glossary

**Table:**

6-MP	6-mercaptopurine
6-TG	6-thioguanine
36B4 (RPLP0)	ribosomal protein lateral stalk subunit P0
AIP	autoimmune pancreatitis
ANO1	anoctamin 1
ANOVA	analysis of variance
ALB	albumin
ALP	alkaline phosphatase
ALT	alanine aminotransferase
ASGR1	asialoglycoprotein receptor 1
AST	aspartate aminotransferase
AZA	azathioprine
BCA	bicinchoninic acid
BCECF AM	2′ 7′-bis-(2-carboxyethyl)-5-(and-6)-carboxyfluorescein acetoxymethyl ester
BCO	bile-collected cholangiocyte organoid
BSA	bovine serum albumin
CA19.9	cancer antigen 19.9
CCA	cholangiocarcinoma
Cq	cycle quantification
CRP	C-reactive protein
DEAE	diethylaminoethyl
DEPC	diethylpyrocarbonate
DPMI	disintegrations per minute
DTT	dithiothreitol
ECM	extracellular matrix
ECO	extrahepatic cholangiocyte organoid
EDTA	ethylenediaminetetraacetic acid
ELISA	enzyme-linked immunosorbent assay
EPCAM	epithelial cell adhesion molecule
ERCP	endoscopic retrograde cholangiopancreatography
ESR	erythrocyte sedimentation rate
GAPDH	glyceraldehyde-3-phosphate dehydrogenase
GCDC	glycochenodeoxycholic acid
GEO	Gene Expression Omnibus
gGT	gamma-glutamyl transferase
GIST	gastrointestinal stromal tumor
HBSS	Hank’s balanced salt solution
HEPES	4-(2-hydroxyethyl)-1-piperazineethanesulfonic acid
HISORt	Histology, imaging, serology, other organ involvement, response to therapy
HPB	hepatopancreatobiliary
HPF	high-power field
HPRT	hypoxanthine phosphoribosyltransferase 1
HRP	horseradish peroxidase
ICO	intrahepatic cholangiocyte organoid
IHC	immunohistochemistry
IgG4-RD	IgG4-related disease
IRC	IgG4-related cholangitis
KD	knockdown
kDa	kilodalton
KRT19	cytokeratin 19
LC-MS/MS	liquid chromatography with tandem mass spectrometry
LGALS3	galectin-3
MMF	mycophenolate mofetil
mRNA	messenger ribonucleic acid
N0	starting concentration
ns	not significant
PBC	primary biliary cholangitis
PBS	phosphate-buffered saline
PEI	polyethylenimine
PHB1	prohibitin 1
PHB2	prohibitin 2
pHi	intracellular pH
PSC	primary sclerosing cholangitis
PTC	percutaneous transhepatic cholangiography
RIPA	radioimmunoprecipitation assay
RPL22	ribosomal protein L22
RT-qPCR	real-time quantitative polymerase chain reaction
SDS-PAGE	sodium dodecyl sulfate–polyacrylamide gel electrophoresis
shRNA	short hairpin ribonucleic acid
TMB	3, 3′, 5, 5′-tetramethylbenzidine
TBS	tris-buffered saline
TBST	tris-buffered saline tween
TCR	T-cell receptor
Th17	T helper 17 cell
TRC	the RNAi consortium
UDCA	ursodeoxycholic acid
WT	wild type
