# Supplementary material for: Galectin-3 and prohibitin 1 are autoantigens in IgG4-related cholangitis without clear-cut protective effects against toxic bile acids
Source: Front Immunol. 2024 Jan 25;14:1251134. doi: 10.3389/fimmu.2023.1251134 (PMC10851949; doi:10.3389/fimmu.2023.1251134)
Supplement: Supplementary file 1 [file DataSheet_1.pdf]

Supplementary data

Title:

Galectin-3 and prohibitin 1 are autoantigens in IgG4-related cholangitis without clear-cut protective effects against toxic bile acids

List of authors:

Remco Kersten[1#], David C. Trampert[1#], Lowiek M. Hubers[1], Dagmar Tolenaars[1], Harmjan R. Vos[2], Stan F.J. van de Graaf[1], Ulrich Beuers[1*]

[#] These authors contributed equally to this work and share first authorship

1. Tytgat Institute for Liver and Intestinal Research, Department of Gastroenterology and Hepatology, Amsterdam Gastroenterology Endocrinology Metabolism (AGEM), Amsterdam UMC, University of Amsterdam, Amsterdam, The Netherlands.
2. Oncode Institute and Molecular Cancer Research, Center for Molecular Medicine, University Medical Center Utrecht, Utrecht, The Netherlands.

*Address for correspondence:
Prof. Dr. Ulrich Beuers
Department of Gastroenterology and Hepatology
Tytgat Institute for Liver and Intestinal Research
Amsterdam UMC, University of Amsterdam, location AMC (C2-327)
Meibergdreef 9, 1105 AZ Amsterdam, The Netherlands
Phone: +31 20 5662422
Fax: +31 20 566 9701
E-mail: u.h.beuers@amsterdamumc.nl

Table of contents:

1. Supplementary methods
2. Supplementary figure S1: Correlation between liver tests- and anti-galectin-3 IgG, anti-galectin-3 IgG1 or anti-galectin-3 IgG4 titers
3. Supplementary figure S2: Correlation between liver tests - and anti-prohibitin 1 IgG, anti-prohibitin 1 IgG1 or anti-prohibitin 1 IgG4 titers
4. Supplementary figure S3: IgG4 autoantibody positivity against annexin A11 determined by ELISA
5. Supplementary figure S4: Serum liver tests over time of patients with anti-galectin-3 and/or anti-prohibitin 1 autoantibodies
6. Supplementary figure S5: Intracellular pH measurements in *LGALS3* KD, GB1107 and recombinant galectin-3 treated H69 cholangiocytes
7. Supplementary figure S6: Intracellular pH measurements in *PHB1*, *PHB2* and combined *PHB1/2* KD H69 cholangiocytes
8. Supplementary figure S7: Intracellular pH measurements in rocaglamide and recombinant prohibitin 1 treated H69 cholangiocytes
9. Supplementary table S1: Human H69 cholangiocyte medium composition
10. Supplementary table S2: RT-qPCR primer and shRNA sequences
11. Supplementary table S3: List of primary and secondary antibodies
12. Supplementary table S4: Clinical characteristics of anti-galectin-3 positive patients
13. Supplementary table S5: Clinical characteristics of anti-prohibitin 1 positive patients
14. Supplementary table S6: Homemade HBSS formulation used for BCECF AM experiments
15. Supplementary table S7: List of chemicals and compounds
16. Supplementary references

Supplementary methods:

Correlation between serum liver tests and serum IgG4, serum IgG1, and IgG4/IgG1 autoantibody titers

ELISAs for anti-galectin-3 and anti-prohibitin 1 positivity were performed as described in the main manuscript. Serum liver tests (bilirubin, alkaline phosphatase, gGT) were extracted from patients' clinical files at the time when patients tested positive for anti-galectin-3 of anti-prohibitin 1. Data of serum liver tests were subsequently assessed for normality distribution using the Kolmogorov-Smirnov test. Correlations were performed by Spearman's rank (Rs) correlation (for non-normally distributed data) or Pearson (R) correlation (for normally distributed data). R^2 values are provided when data was normally distributed. P-values <0.05 were considered statistically significant.

Clinical characteristics of anti-galectin-3 and anti-prohibitin 1 positive patients

For patients who were positive for anti-galectin-3 and/or anti-prohibitin 1 autoantibodies, detailed clinical characteristics were extracted from patients' clinical files. These parameters included age at onset, time to IRC diagnosis, gender, profession/hobbies with > 1 year of toxic chemical exposure, IgG4-RD organ involvement, other autoantibody positivity, history of major hepatopancreatobiliary surgery (bile duct resection, hemihepatectomy, Whipple procedure), history of malignancy and IgG4-RD directed therapy.

Primer design and validation

Primers were self-designed to cover all transcript variants of the gene of interest. To achieve this, FASTA sequences were obtained of all transcript variants and aligned by nucleotide count using a Multiple Sequence Alignment tool (https://www.ebi.ac.uk/Tools/msa/clustalo/). NCBI PRIMER-BLAST was used to design primers restricted to the sequence range identical for all transcript variants (https://www.ncbi.nlm.nih.gov/tools/primer-blast/).

Intracellular pH measurement by 2',7'-bis-(2-carboxyethyl)-5-(and-6)-carboxyfluorescein (BCECF)

This assay was largely performed as previously described (1). H69 cholangiocytes were plated in 96-well black solid flat bottom tissue culture treated plates (Corning #3916) and grown until confluency. A working solution of 5 µM BCECF acetoxymethylester (AM) (Invitrogen #B1150) was prepared in 20 mM HEPES-buffered Hank's Balanced Salt Solution (HBSS) (Lonza #BE10-527F, Basel, Switzerland) pH 7.4 without phenol red. After a series of washing steps with HEPES-buffered HBSS, cells were loaded with BCECF AM for 45 minutes at room temperature and protected from light. Three wells per condition or cell line were left unloaded for blank measurements. Excessive dye was removed, and the plate was incubated for an additional 30 minutes in HEPES-buffered HBSS.

A pH standard was prepared from a high-potassium Nigericin (10 µM) solution supplemented with 30 mM HEPES spanning over the physiological pH range. BCECF AM loaded wells were filled with the pH standards and experimental solutions were added to the loaded and blank wells of each condition. The plate was placed in the CLARIOstar (BMG LABTECH, Ortenberg, Germany) set at 37 ºC and a gain adjustment for all wells was performed aiming for 50% of maximal fluorescent values. Ratiometric fluorescent measurement was performed at dual excitation wavelength: $\lambda Ex1$=490-10 nm (pH-sensitive) / $\lambda Ex2$=440-10 nm (pH-insensitive), emission was collected at λEm=535-20 nm. pH standards were measured until a steady state was acquired. After this, experimentally treated wells were measured at 37 ºC with 5% CO_2 inflow until a steady intracellular pH state was acquired.

Raw data were converted to fluorescent ratios after background fluorescence from the unloaded blank wells were subtracted at all time points. The fluorescent ratio R was calculated as F490/440. Fluorescent ratios were converted to pH using the fluorescent ratios from the high-potassium Nigericin pH standards. This was done by performing nonlinear least-square fitting according to the following equation as previously described (2-3): $pH = pK_a - \log\frac{R- R_A}{R_B - R} \times \frac{F_{A(\lambda 2)}}{F_{B(\lambda 2)}}$ where R stands for F490/440, RA and RB represent fluorescent ratios at extreme acidic and basic conditions respectively, $F_{A(\lambda 2)}$ and $F_{B(\lambda 2)}$ are the fluorescent values λEx2 440 nm at extreme acidic and basic conditions respectively. Baseline intracellular pH values were determined by averaging the intracellular pH values of the last 8 time points. Experimental solutions were made in homemade HBSS (see supplementary table S6). Experimental conditions tested in BCECF experiments: GB1107; 10 µM, 24 hours pretreatment. Recombinant galectin-3; 2.5 µg/ml, 24 hours pretreatment. Rocaglamide; 100 nM, 24 hours pretreatment. Recombinant prohibitin 1; 0.25 µg/ml and 0.5 µg/ml, 24 hours pretreatment.

Figure S1: Correlation between serum liver tests and anti-galectin-3 IgG, anti-galectin-3 IgG1 or anti-galectin-3 IgG4 titers.

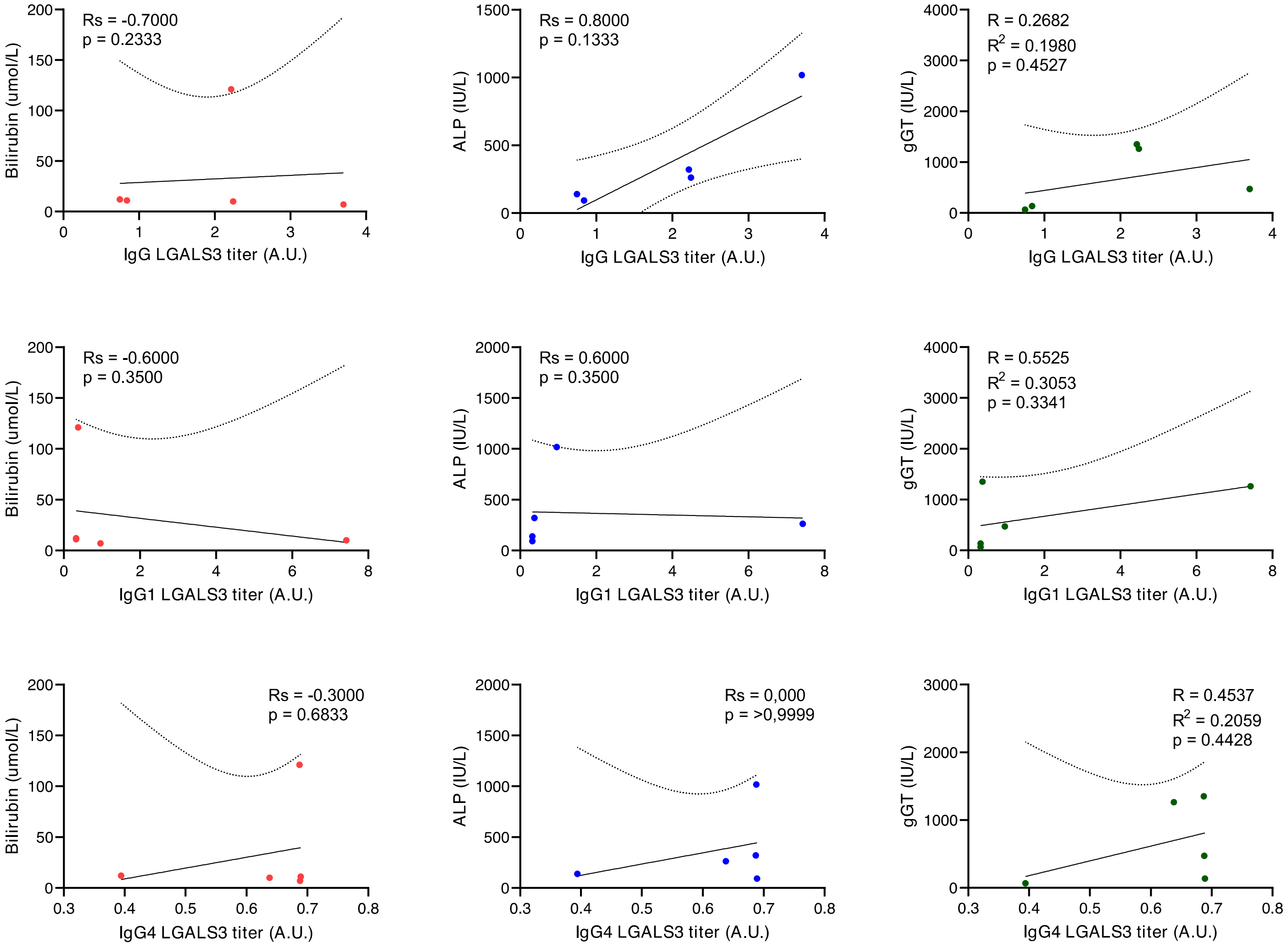

Figure S1: Correlations between serum liver tests and anti-galectin-3 IgG/IgG1/IgG4 titers as determined by optical density measurements at 450nm. Abbreviations: ALP, alkaline phosphatase; gGT, gamma glutamyl-transferase; OD, optical density; R, Pearson correlation coefficient; R^2, coefficient of determination; Rs, Spearman's rank correlation coefficient.

Figure S2: Correlation between serum liver tests and anti-prohibitin 1 IgG, anti-prohibitin 1 IgG1 or anti-prohibitin 1 IgG4 titers.

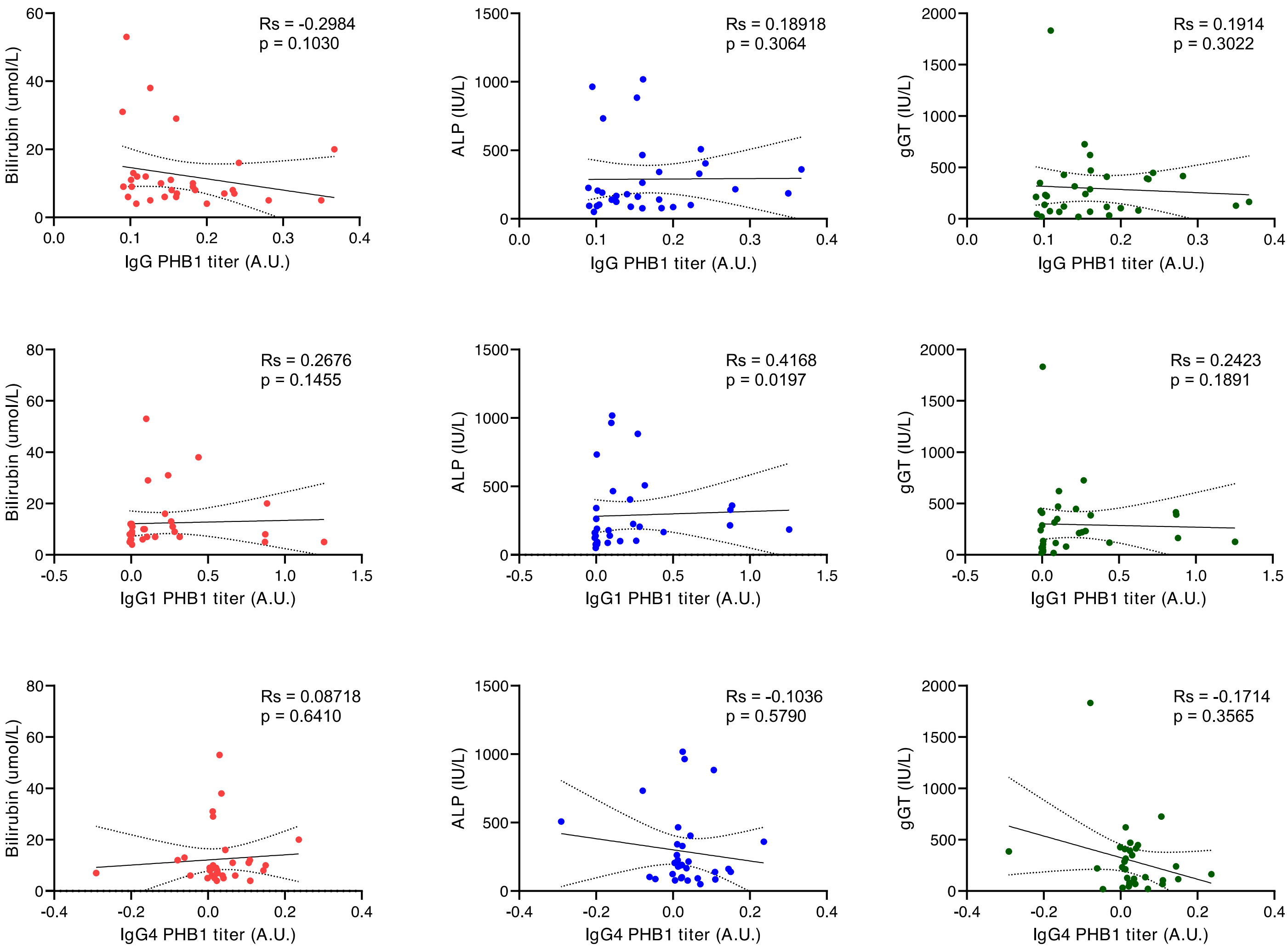

Figure S2: Correlations between serum liver tests and anti-prohibitin 1 IgG/IgG1/IgG4 titers as determined by optical density measurements at 450nm. Abbreviations: ALP, alkaline phosphatase; gGT, gamma glutamyl-transferase; OD, optical density; R, Pearson correlation coefficient; R^2, coefficient of determination; Rs, Spearman's rank correlation coefficient.

Figure S3: IgG4 autoantibody positivity against annexin A11 determined by ELISA.

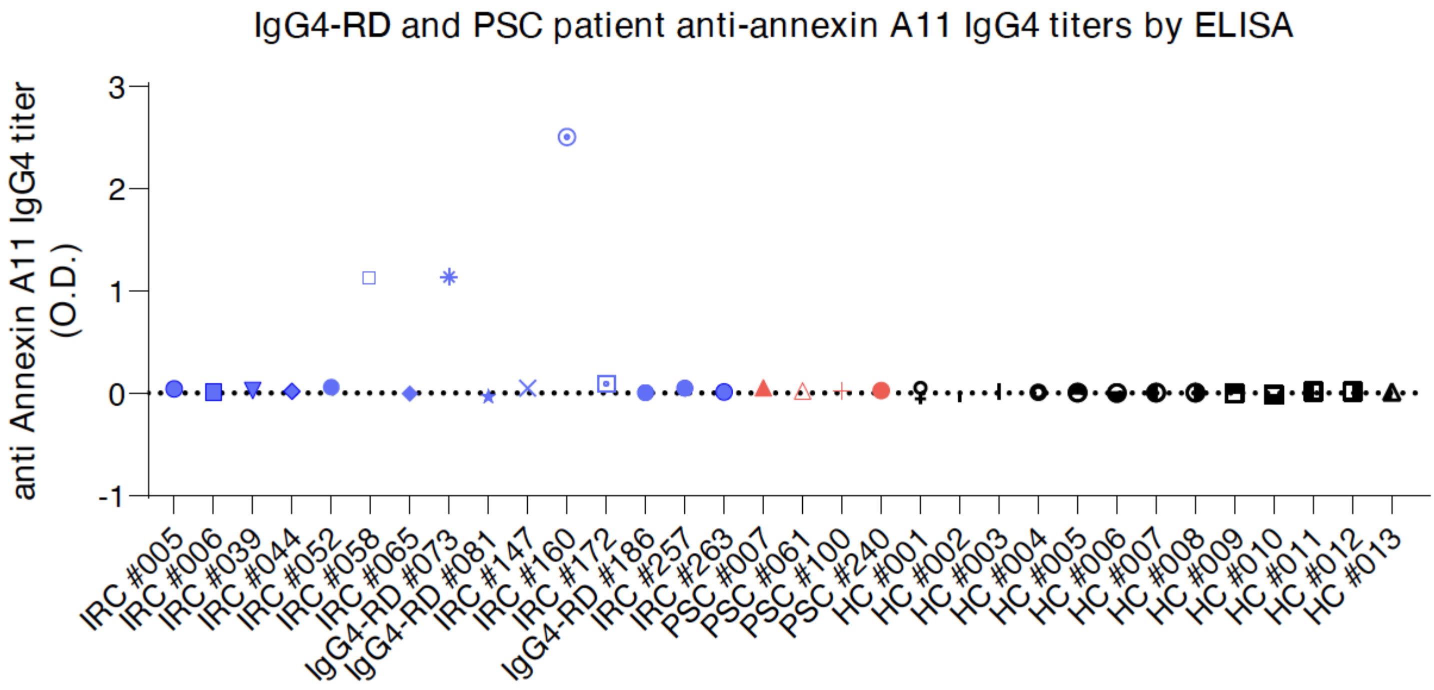

Figure S3: Anti-annexin A11 IgG4 titers in people with IRC, IgG4-RD, PSC and healthy controls. Data are represented as individual data points. Abbreviations: ELISA, enzyme-linked immunosorbent assay; IgG4-RD, IgG4-related disease; IRC, IgG4-related cholangitis; PSC, primary sclerosing cholangitis; O.D., optical density.

Figure S4: Serum liver tests over time of patients with anti-galectin-3 and/or anti-prohibitin 1 autoantibodies.

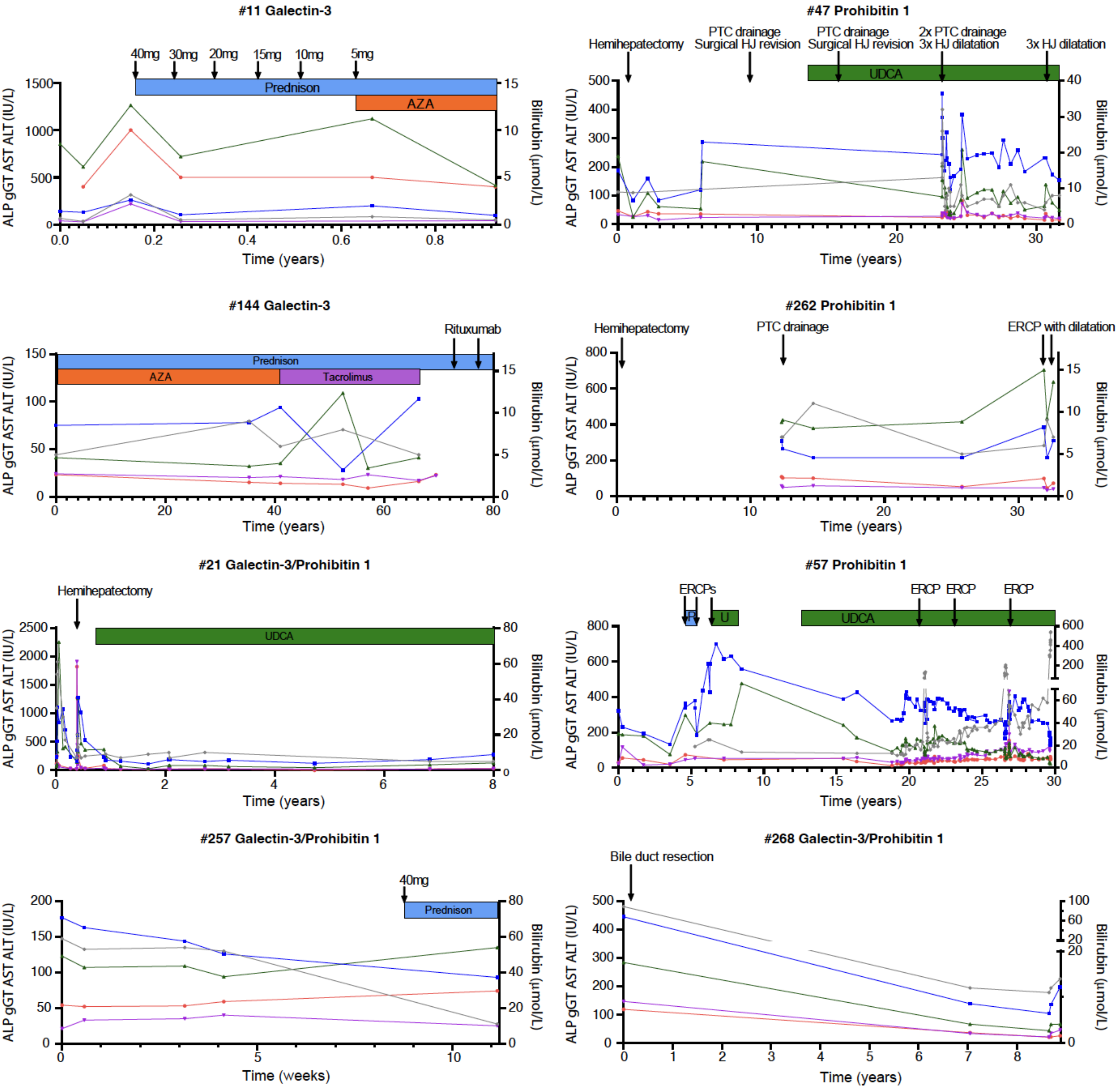

Figure S4: Serum liver tests over time of patients positive for anti-galectin-3 and/or anti-prohibitin 1 autoantibodies. Patients are annotated in figure 2 A-F. Abbreviations: ALP, alkaline phosphatase; ALT, alanine aminotransferase; AST, aspartate aminotransferase; AZA, azathioprine; ERCP, endoscopic retrograde cholangiopancreaticography; gGT, gamma glutamyl-transferase; HJ, hepaticojejunostomy; PTC, percutaneous transhepatic cholangiography; UDCA, ursodeoxycholic acid.

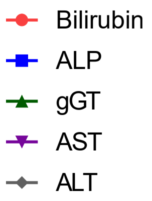

Figure S5: Intracellular pH measurements in *LGALS3* KD, GB1107 and recombinant galectin-3 treated H69 cholangiocytes.

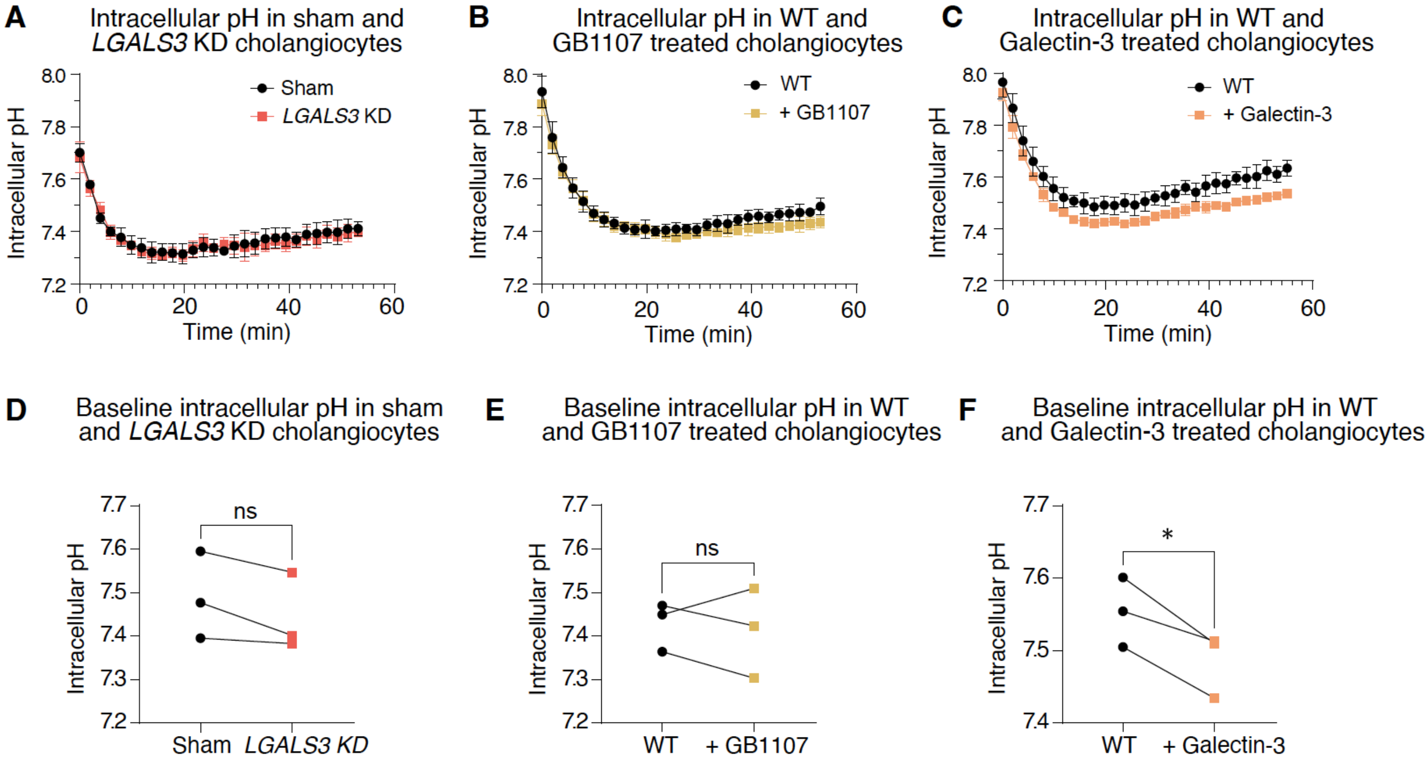

Figure S5: Intracellular pH tracing in (**A**) sham and *LGALS3* KD cholangiocytes, (**B**) WT and GB1107 treated cholangiocytes, and (**C**) WT and recombinant galectin-3 treated cholangiocytes (representative experiment of n=3). Quantification of baseline intracellular pH of (**D**) sham and *LGALS3* KD cholangiocytes, (**E**) WT and GB1107 treated cholangiocytes and (**F**) WT and recombinant galectin-3 treated cholangiocytes (3 average baseline values from n=3 independent experiments). Levels of significance: (**D**) ns, not significant p=0.1301, paired t-test. (**E**) ns, p=0.7202, paired t-test. (**F**) * p=0.0429, paired t-test. Abbreviations: KD, knockdown; *LGALS3*, galectin-3; ns, not significant; WT, wild type.

Figure S6: Intracellular pH measurements in *PHB1*, *PHB2* and combined *PHB1/2* KD H69 cholangiocytes.

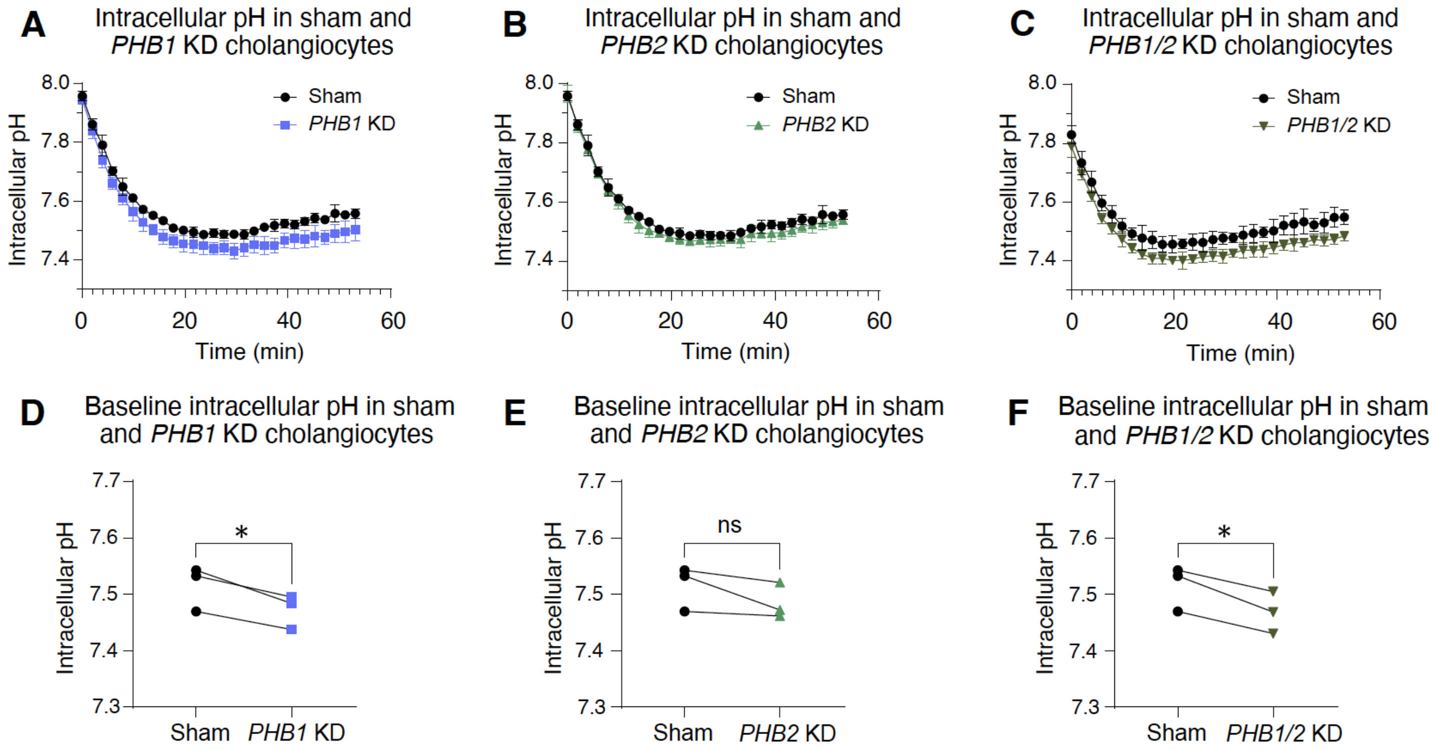

Figure S6: Intracellular pH tracing in (**A**) sham and *PHB1* KD cholangiocytes, **(B)** sham and *PHB2* KD cholangiocytes, (**C**) sham and combined *PHB1/2* KD cholangiocytes. Quantification of baseline intracellular pH of (**D**) sham and *PHB1* KD cholangiocytes, (E) sham and *PHB2* KD cholangiocytes, (**F**) sham and combined *PHB1/2* KD cholangiocytes. Levels of significance: **(D)** * p=0.0337, **(E)** ns, not significant p=0.1960, **(F)** * p=0.321, paired t-tests. Abbreviations: KD, knockdown; ns, not significant; *PHB1*, prohibitin 1; *PHB2*, prohibitin 2; WT, wild type.

Figure S7: Intracellular pH measurements in rocaglamide and recombinant prohibitin 1 treated H69 cholangiocytes.

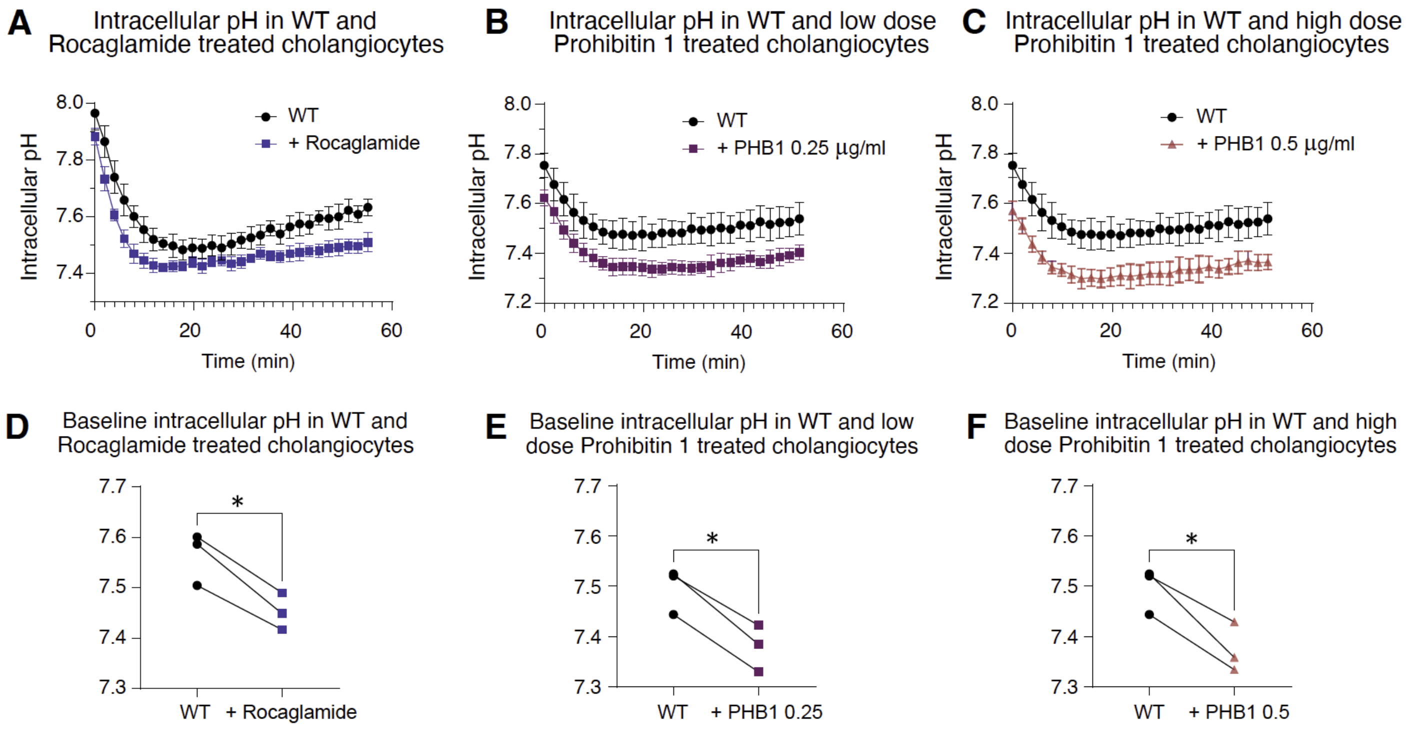

Figure S7: Intracellular pH tracing in (**A**) WT and rocaglamide treated cholangiocytes, (**B**) WT and recombinant low-dose prohibitin 1 0.25 µg/ml treated cholangiocytes and (**C**) WT and recombinant high-dose prohibitin 1 0.5 µg/ml treated cholangiocytes (representative experiment of n=3). Quantification of baseline intracellular pH of (**D**) WT and rocaglamide treated cholangiocytes, (**E**) WT and recombinant low-dose prohibitin 1 0.25 µg/ml treated cholangiocytes and (**F**) WT and recombinant high-dose prohibitin 1 0.5 µg/ml treated cholangiocytes (3 average baseline values from n=3 independent experiments). Levels of significance: **(D)** * p=0.0160, **(E)** * p=0.0107, **(F)** * p=0.0320, paired t-tests. Abbreviations: KD, knockdown; ns, not significant; PHB1, prohibitin 1; WT, wild type.

Table S1: Human H69 cholangiocyte medium composition.

Material / Compound	Cat. No.	Supplier	Volume/Concentration
DMEM	31600083	Gibco	375 mL
Ham's F-12 Nutrient Mix	N6760	Sigma	125 mL
Sodium Bicarbonate	144-55-8	Merck	1.794 g/L
HEPES-NaOH, pH 7.4	H3375	Sigma	20 mM
L-glutamine	BE-17-605E	Lonza	3.25 mM
Penicillin/Streptomycin	15140-122	Gibco	37.5 U/ml / 37.5 µg/ml
Fetal Bovine Serum	S00EW10003<br>Batch: BDC-S00EW1	Bodinco	10% (v/v)
Adenine	A2786	Sigma	180 µM
Insulin	I5523	Sigma	865 nM
Transferrin	T8158	Sigma	62.5 nM
Hydrocortisone	H0888	Sigma	1.1 µM
Epinephrine	E4250	Sigma	5.5 µM
Epidermal Growth Factor	E9644	Sigma	1.67 nM
Triiodothyronine (T3)	T3697	Sigma	2 nM

Table S1: Composition of human H69 cholangiocyte medium with corresponding catalogue number, supplier and volume/concentration used.

Table S2: RT-qPCR primer and shRNA sequences.

Gene target	Primer sequence Fw	Primer sequence Rv	shRNA TRC#	shRNA sequence	MISSION shRNA KD library validation
LGALS3	TGGAGCACCTGGAGCTTATC	GGTTATAAGGCACAATCAGTGGC	TRCN0000029305	CCCACGCTTCAATGAGAACAA	96%
PHB1	CATGTGCGGCTGAACTGAGA	TTCCGCTGGTCACACAGTTA	TRCN0000029207	GCTGCCGTCCATCACAACTGA	97%
PHB2	CACCAGGGGAAGTGACAGCC	ATGATACTGGGGCGGGGTAG	TRCN0000060922	CCTAGCATGTACCAGCGCCTA	78%
HPRT	CCTGGCGTCGTGATTAGTGAT	AGACGTTCAGTCCTGTCCATAA	-	-	-
RPLP0 (36B4)	TCATCAACGGGTACAAACGA	GCCTTGACCTTTTCAGCAAG	-	-	-

Table S2: List of primers and shRNA constructs used for RT-qPCR experiments and generation of stable H69 knockdown cell lines. Abbreviations: Fw, forward; *HPRT*: hypoxanthine phosphoribosyltransferase 1; *LGALS3*, galectin-3; *PHB1*, prohibitin 1; *PHB2*, prohibitin 2; *RPLP (36B4)*, ribosomal protein lateral stalk subunit P0; Rv, reverse.

Table S3: List of primary and secondary antibodies

Name	Species	Dilution	Supplier	Cat no.	Application
Galectin-3	Mouse	1:1000 /1:100	Santa Cruz	sc-32790	WB/IHC
Prohibitin 1	Mouse	1:1000 /1:100	Santa Cruz	sc-377037	WB/IHC
Cytokeratin 19	Rabbit	1:100	Abcam	ab15463	IHC
GAPDH	Rabbit	1:5000	Cell Signaling	#2118	WB
Beta-actin	Mouse	1:5000	Sigma	A5441	WB
Bright vision anti-rabbit/mouse HRP	Goat	Ready-to-use	Immunologic	VWRKDPVO110HRP	IHC
Anti-mouse HRP	Goat	1:2000	Biorad	170-6516	WB
Anti-rabbit HRP	Goat	1:2000	Biorad	170-6515	WB
Anti-human IgG HRP	Rabbit	1:2000	Dako	P0214	ELISA
Anti-human IgG1 HRP	Mouse	1:2000	Southern Biotech	#9052-05	ELISA
Anti-human IgG4 HRP	Mouse	1:2000	Southern Biotech	#9200-05	ELISA

Table S3: List of primary and secondary antibodies used for western blotting, immunohistochemistry and ELISA. Abbreviations: ELISA, enzyme-linked immunosorbent assay; GAPDH, Glyceraldehyde-3-phosphate dehydrogenase; HRP, Horse radish peroxidase; IHC, immunohistochemistry; WB, western blot.

Table S4: Clinical characteristics of anti-galectin-3 positive patients.

	#21	#11	#148	#144	#160	#257	#268
**Age at onset** (years)	66	43	49	71	UNK	53	74
**Time to diagnosis** (months)	11	72	5	0	UNK	114	93
**Gender**	M	M	M	M	M	M	M
**Profession/Hobbies**	Painting	Carpenter	Road construction worker	Painter	UNK	Metal worker	UNK
**Organ involvement**	IRC<br>AIP<br>Prostate	IRC<br>AIP<br>Orbital<br>Submandibular glands<br>Nasal Polyps	IRC<br>AIP	IRC<br>AIP	IRC<br>AIP<br>Prostate<br>Kidney	IRC<br>AIP<br>Lacrimal gland<br>Submandibular gland	IRC
**Autoantibody positivity**	Galectin-3<br>Prohibitin 1	Annexin A11<br>Galectin-3	Galectin-3	Galectin-3	Annexin A11<br>Galectin-3<br>Prohibitin 1	Galectin-3<br>Prohibitin 1	Galectin-3<br>Laminin 511-E8<br>Prohibitin 1
**Galectin-3 IgG positivity SD**	3SD	3SD	3SD	2SD	3SD	2SD	2SD
**HPB surgery**	Hemihepatectomy	No	Hemihepatectomy	No	UNK	No	Bile duct resection
**Malignancy**	Sigmoid carcinoma<br>(40 $IgG4^+$ cells/HPF)	No	No	Prostate carcinoma	UNK	No	No
**Therapy**	PTC drainage<br>UDCA	Prednisone<br>Azathioprine	UDCA<br>Prednisone<br>PTC drainage	Prednisone<br>Azathioprine<br>Tacrolimus<br>Rituximab	UNK	Prednisone<br>Azathioprine	No

Table S4: Clinical characteristics of individual patients positive for anti-galectin-3 autoantibodies. Patient numbers per column match patient numbers in Figure 1A-C of the main manuscript. Abbreviations: AIP, autoimmune pancreatitis type 1; F, female; HPF, high-power field; IRC, IgG4-related cholangitis; M, male; PTC, percutaneous transhepatic cholangiography; UDCA, ursodeoxycholic acid; UNK, unknown.

Table S5: Clinical characteristics of anti-prohibitin 1 positive patients.

Patient	Age at onset (years)	Time to diagnosis (months)	Gender	Profession /Hobbies	Organ involvement	Autoantibody positivity	Prohibitin 1 IgG positivity SD	HPB Surgery	Malignancy	Therapy
**#75**	56	108	M	Instrument maker	IRC	Annexin A11<br>Prohibitin 1	3SD	No	Acute myeloid leukemia	Prednisone<br>UDCA<br>Azathioprine<br>MMF<br>ERCP with stent
**#87**	68	7	M	Computer technician	IRC, AIP, Aorta	Prohibitin 1	2SD	No	No	Prednisone<br>AZA<br>ERCP with stent
**#9**	67	36	M	Carpenter	IRC<br>AIP<br>Kidney	Prohibitin 1	3SD	No	Bladder carcinoma<br>Melanoma	Prednisone<br>UDCA<br>Azathioprine<br>ERCP with stent
**#47**	41	279	F	Teacher<br>(pesticide exposure)	IRC	Prohibitin 1	3SD	Hemihepatectomy	Mamma carcinoma	PTC drainage<br>UDCA
**#44**	69	12	M	Car mechanic	IRC	Prohibitin 1	3SD	Hemihepatectomy	Bladder carcinoma<br>GIST	ERCP with stent<br>PTC drainage<br>Prednisone<br>Azathioprine<br>UDCA
**#50**	71	2	M	Greenhouse worker	IRC	Prohibitin 1	2SD	No	Cholangiocarcinoma	ERCP with stent<br>PTC drainage<br>Prednisone<br>Azathioprine<br>MMF
**#24**	75	60	M	Baker	IRC<br>AIP<br>Laryngeal	Annexin A11<br>Prohibitin 1	3SD	No	No	ERCP with stent
**#53**	58	11	M	Construction worker	IRC<br>AIP	Annexin A11<br>Prohibitin 1	3SD	Hemihepatectomy	No	ERCP with stent<br>Prednisone<br>UDCA
**#65**	66	1	M	UNK	IRC<br>AIP	Prohibitin 1	3SD	No	Kidney malignancy	ERCP with stent<br>Prednisone<br>Azathioprine
**#42**	52	4	M	Truck driver	IRC	Annexin A11<br>Laminin 511-E8<br>Prohibitin 1	3SD	No	No	Spontaneous improvement
**#67**	69	1	M	Metal worker	IRC<br>Kidney	Annexin A11<br>Prohibitin 1	3SD	No	No	ERCP with stent<br>Prednisone<br>Azathioprine
**#157**	42	6	M	UNK	IRC<br>AIP<br>Pulmonary	Prohibitin 1	2SD	No	No	ERCP with stent<br>Prednisone<br>Azathioprine
**#21**	66	11	M	Painting	IRC<br>AIP<br>Prostate	Galectin-3<br>Prohibitin 1	3SD	Hemihepatectomy	Sigmoid carcinoma<br>(40 IgG4+ cells/HPF)	PTC drainage<br>UDCA
**#177**	72	1	M	Financial management	IRC	Prohibitin 1	2SD	No	No	Prednisone<br>Azathioprine<br>6-MP
**#223**	63	24	M	Communication advisor	IRC<br>AIP	Prohibitin 1	3SD	Whipple	Liver tumor	Budesonide<br>UDCA<br>Bezafibrate
**#225**	63	6	M	Carpenter<br>Construction worker	IRC<br>Submandibular glands	Annexin A11<br>Prohibitin 1	3SD	Hemihepatectomy	No	PTC drainage<br>Prednisone<br>Azathioprine<br>6-MP<br>6-TG<br>UDCA

Patient	Age at onset (years)	Time to diagnosis (months)	Gender	Profession /Hobbies	Organ involvement	Autoantibody positivity	Prohibitin 1 IgG positivity SD	HPB Surgery	Malignancy	Therapy
**#233**	63	4	M	Prison guard	IRC<br>AIP	Prohibitin 1	3SD	No	No	Prednisone
**#160**	66	UNK	M	UNK	IRC<br>AIP<br>Kidney<br>Prostate	Annexin A11<br>Galectin-3<br>Prohibitin 1	2SD	UNK	UNK	Prednisone
**#80**	71	8	M	Journalist	IRC<br>Pulmonary	Prohibitin 1	3SD	No	Esophageal carcinoma	UDCA
**#95**	55	108	M	Construction worker	IRC	Prohibitin 1	3SD	No	No	Prednisone<br>6-TG<br>UDCA
**#57**	34	360	F	Cleaner	IRC<br>AIP<br>Pulmonary<br>Colon	Annexin A11<br>Laminin 511-E8<br>Prohibitin 1	3SD	No	Cholangiocarcinoma	ERCP with stent<br>Prednisone<br>UDCA
**#52**	50	252	M	Construction worker	IRC<br>AIP	Prohibitin 1	3SD	Bile duct resection	No	PTC drainage<br>Prednisone<br>Azathioprine<br>UDCA
**#51**	63	9	M	Military service<br>Limousine driver	IRC	Laminin 511-E8<br>Prohibitin 1	2SD	Hemihepatectomy	Colorectal carcinoma	UDCA
**#269**	56	12	M	Construction worker	IRC	Prohibitin 1	3SD	Bile duct resection	No	ERCP with stent<br>UDCA
**#270**	51	13	M	Firefighter<br>Car mechanic	IRC	Prohibitin 1	3SD	Bile duct resection	No	Prednisone<br>Azathioprine<br>UDCA
**#288**	65	10	M	Construction worker	IRC<br>AIP	Laminin 511-E8<br>Prohibitin 1	3SD	Bile duct resection	Sigmoid carcinoma	PTC drainage<br>Prednisone<br>6-TG<br>UDCA
**#262**	23	312	M	Car mechanic<br>Construction worker	IRC	Prohibitin 1	3SD	Hemihepatectomy	No	PTC drainage<br>UDCA
**#271**	55	147	F	Secretary	IRC	Prohibitin 1	3SD	Hemihepatectomy	No	ERCP with stent<br>Prednisone<br>UDCA
**#28**	50	36	M	UNK	IRC<br>AIP	Prohibitin 1	2SD	No	No	Prednison<br>Azathioprine<br>UDCA
**#74**	42	2	F	Beautician<br>Car sales	IRC	Laminin 511-E8<br>Prohibitin 1	2SD	Hemihepatecomy<br>PPPD	Cholangiocarcinoma	Prednison<br>Budesonide<br>Azathioprine<br>6-TG<br>UDCA
**#257**	53	114	M	Metal worker	IRC<br>AIP<br>Lacrimal glands<br>Submandibular glands	Galectin-3<br>Prohibitin 1	2SD	No	No	Prednison<br>Azathioprine
**#268**	74	93	M	UNK	IRC	Galectin-3<br>Laminin 511-E8<br>Prohibitin 1	3SD	Bile duct resection	No	Bile duct resection

Table S5: Clinical characteristics of individual patients positive for anti-prohibitin 1 autoantibodies. Abbreviations: 6-MP, mercaptopurine; 6-TG, thioguanine; AIP, autoimmune pancreatitis type 1; ERCP, Endoscopic retrograde cholangiopancreaticography; F, female. GIST, gastrointestinal stromal tumor; IRC, IgG4-related cholangitis; M, male; MMF, mycophenolate mofetil; PPPD, pylorus preserving pancreatoduodenectomy; PTC, percutaneous transhepatic cholangiography; UDCA, ursodeoxycholic acid; UNK, unknown.

Table S6: Homemade HBSS formulation used for BCECF AM experiments.

Component	Final concentration (mM)	Osmolarity mosm/L)
$CaCl_2$ * $2H_2O$	1.27	3.80
KCl	4.56	9.12
NaH_2PO_4 * $2H_2O$	0.45	0.90
$MgSO_4$ * $7H_2O$	0.41	0.81
$MgCl_2$ * $6H_2O$	0.39	1.18
NaCl	107.80	215.61
$NaHCO_3^-$	21.43	42.85
Na_2HPO_4 * $2H_2O$	0.34	1.01
HEPES-NaOH, pH 7.4	20	30
Glucose	5.55	5.55

Table S6: Composition of homemade HBSS used in BCECF AM experiments.

Table S7: List of chemicals and compounds.

Material / Compound	Cat. No	Supplier
Complete^{TM} Protease Inhibitor Cocktail	11697498001	Roche
Pierce^{TM} BCA Protein Assay Kit	23225	Thermo Scientific
Lumi-light	12015196001	Roche
BCECF-AM	B1170	Thermo Scientific
HBSS phenol red free	10-527F	Lonza
Glycochenodeoxycholate	G0759	Sigma-Aldrich
22,23-^{3}H-sodium glycochenodeoxycholate	-	Dr. Alan Hofmann
Fatty-acid free BSA	A6003	Sigma-Aldrich
Digitonin	3043	Merck
Recombinant galectin-3	450-38	Peprotech
Recombinant prohibitin 1	P5113	FineTest
3,3′,5,5′- tetramethylbenzidine (TMB)	CL07	Merck
Stop solution	N600	Thermo Scientific
TRIzol	T9424	Sigma
Chloroform	2445	Merck
Isopropyl alcohol	1040	Merck
Diethylpyrocarbonate (DEPC)	D5757	Sigma
DNase I	M6101	Promega
Random Hexamer primers	SO142	Promega
Revertaid transcriptase	EP0442	Fermentas
Nigericin (sodium salt)	11437	Cayman Chemical
SensoLyte Homogeneous Rh110 Caspase-3/7 Assay Kit	AS-71141	AnaSpec
Ultra V Blocking	TA-125-UB	Thermo Scientific
Vectamount Permanent Mounting Medium	H-5000	Vector Laboratories
Sodium citrate	#C9999	Sigma-Aldrich
Raptinal	AG-CR1-2902-M005	AdipoGen Life Sciences
Rocaglamide	SML0656-100UG	Sigma
GB1107	HY-114409	MedChemExpress
Recombinant annexin A11	ab10150	Abcam
Hydrogen peroxide	#8.22287.1000	VWR
PureProteome Protein A/G Mix Magnetic beads	LSKMAGAG10	Millipore

Table S7: Chemicals and compounds used with corresponding catalogue number and supplier.

Supplementary references

1. Chang JC, Go S, de Waart DR, Munoz-Garrido P, Beuers U, Paulusma CC, et al. Soluble Adenylyl Cyclase Regulates Bile Salt-Induced Apoptosis in Human Cholangiocytes. Hepatology. 2016;64(2):522-34.
2. Brown AM. A step-by-step guide to non-linear regression analysis of experimental data using a Microsoft Excel spreadsheet. Comput Methods Programs Biomed. 2001;65(3):191-200.
3. Kemmer G, Keller S. Nonlinear least-squares data fitting in Excel spreadsheets. Nat Protoc. 2010;5(2):267-81.
